# Multifunctional materials for catalyst-specific heating and thermometry in tandem catalysis†

**DOI:** 10.1039/d3ta03654e

**Published:** 2023-09-13

**Authors:** Marcos G. Farpón, Raquel Peláez, Verónica Recio, Burak Atakan, Carlos Zaldo, Gonzalo Prieto

**Affiliations:** a ITQ Instituto de Tecnología Química, Universitat Politècnica de València-Consejo Superior de Investigaciones Científicas (UPV-CSIC) Avenida de los Naranjos s/n Valencia 46022 Spain prieto@itq.upv.es; b Max-Planck-Institut für Kohlenforschung Kaiser-Wilhelm-Platz 1 45470 Mülheim an der Ruhr Germany; c Thermodynamik, EMPI, Faculty for Engineering, University of Duisburg-Essen Lotharstr. 1 47057 Duisburg Germany; d Instituto de Ciencia de Materiales de Madrid, Consejo Superior de Investigaciones Científicas CSIC Sor Juana Inés de la Cruz 3 28049 Madrid Spain

## Abstract

A multifunctional material design, integrating catalytic as well as auxiliary magnetic susception and contactless thermal sensing functionalities, unlocks catalyst-specific heating and thermometry for spatially proximate solid catalysts in a single reactor. The new concept alleviates temperature incompatibilities in tandem catalysis, as showcased for the direct production of propene from ethene, *via* sequential olefin dimerization and metathesis reactions.

## Abstract 2

A multifunctional material design, integrating catalytic and auxiliary magnetic susception and contactless thermal sensing functionalities, unlocks catalyst-specific heating and thermometry for spatially proximate solid catalysts in a single reactor.

## Graphical abstract

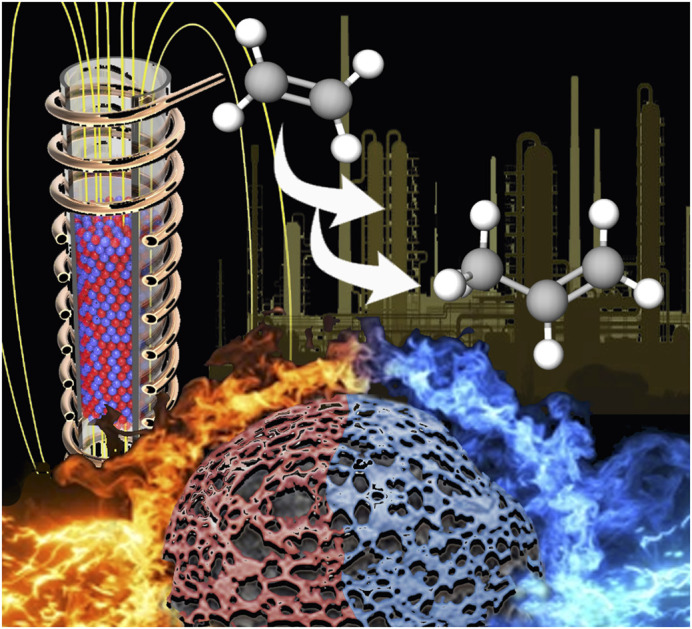

The intensification of chemical processes is considered a crucial stepping-stone in the quest for a more sustainable chemical industry.^1^ The notion of tandem catalysis, *i.e.* the integration of two catalysts to steer sequential and mechanistically decoupled chemical reactions in a single reactor, is a versatile tool which holds a multifold potential to contribute to process intensification.^2–4^ Coupling conversions in a single pot supersedes energy-intensive intermediate workup operations to isolate and purify reaction products. Moreover, the selectivity of the overall transformation may be improved if highly reactive intermediate products, which emerge from a first reaction step, can be intercepted and further converted *in situ* on a proximate second catalyst, minimizing their residence time in the reaction medium and thereby abating undesired decomposition or side-reactions.

Although successfully demonstrated to realize *e.g.* processes for syngas valorization,^5^ alkane oxidation,^6^ and CO_2_ (electro)reduction to multicarbon products,^7,8^ the concept of tandem catalysis faces an enduring barrier towards its full exploitation, *i.e.* the catalysts must share a common reaction medium and reaction settings, which frequently translates into suboptimal individual performance. For many conceivable, yet unrealized, tandem processes a core dichotomy is faced: the integrated catalysts should operate *spatially proximate*, so that transport of intermediate products between their active centers is fast and effective, minimizing the probability for side-reactions, but *thermally distant*, enabling each catalyst to operate within its own temperature window, hence with (close to) optimal individual performance. Herein we introduce a multifunctional material design which enables reconciling short inter-catalyst mass transport distances with individual temperature control for two solid catalysts integrated in a tandem conversion process.

Localized heating *via* dielectric, inductive, Joule or plasmonic effects has been exploited to induce stimuli responsiveness, sensoring and surface catalytic conversions.^9,10^ We capitalize on spatially non-uniform temperature distributions caused by localized magnetic induction heating in a radiofrequency (RF) oscillating field to deliberately establish a temperature gradient between two proximate solid catalysts. Fig. 1a–c summarizes schematically the multifunctional material design proposed. A first catalyst material has been furnished with magnetic susception functionalities, as nanoscale heat sources in a RF field, within a porous matrix of the catalytically active material. The active centers on this *hot catalyst* are meant to operate at a higher temperature in the tandem conversion. A second (*cold*) catalyst is meant to operate at a comparatively lower temperature, lacks magnetic susception functionalities and it is therefore heated mainly *via* convective heat transfer from the fluid circulating in the reactor.

**Fig. 1:**
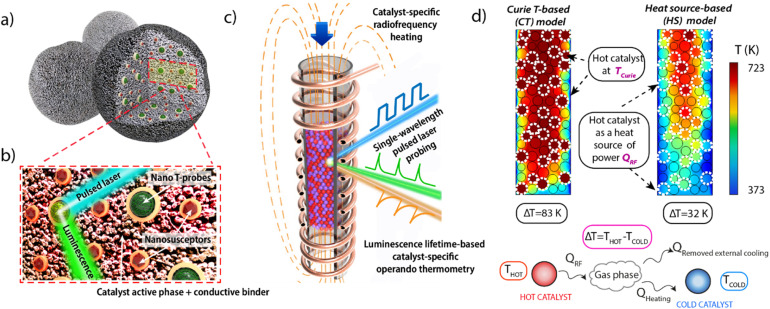
(a) Schematic representation of the multifunctional design of consolidated catalyst bodies incorporating additional auxiliary magnetic susception and thermophosphor sensing functionalities. (b) Blown-up view of the cross-sectional area marked with the red frame in panel (a). Ferromagnetic nanocrystals (brown polyhedra) and lanthanide-based thermophosphor nanoparticles (green, multigrain spheres) appear coated by a continuous SiO_2_ overlay. (c) Schematic depiction of a packed-bed reactor inserted in a RF coil and filled with a blend of consolidated bodies of two catalyst materials between which a steady temperature gradient is stablished by catalyst-specific heating. The first catalyst material features ferromagnetic susceptors and thus self-heats *via* induction mechanisms (red particles). The second “cold” catalyst material lacks ferromagnetic susception and it is mainly heated *via* convective mechanisms (purple particles). Temperature is monitored *via operando* luminescence thermometry (d) Computer Fluid Dynamics (CFD)-derived temperature profiles obtained for a model reactor assuming two scenarios for the heating of the *hot catalyst*. Further experimental details on the CFD simulations are provided in Section 1.3 of the ESI.†

As significant as achieving heating with material specificity is to assess and control temperature under *operando* conditions and with catalyst specificity. Conventional thermometry methods are ambiguous under such conditions, as they typically require the application of metallic probes, which self-heat by Joule effects in a RF field, whilst they lack material specificity. Alternatively, luminescence spectroscopy provides a contactless thermometry technique based on the temperature-sensitive quenching of luminescence in so-called thermophosphor materials.^11,12^ Farpón *et al.*^13^ and Geitenbeek *et al.*^14^ independently introduced this method for *operando* thermometry in packed-bed catalytic reactors, and the concept was later extended to slurry-phase catalysis.^15^ We herein prove this method to be an effective approach to independently probe the operation temperature of two catalyst materials in a tandem conversion process subjected to differential heating.

As shown in Fig. 1, composite catalysts are additionally implanted with nanosized thermophosphor functionalities. The latter can be probed, while at work in a flow quartz reactor, using a UV laser as excitation source. This way, the remote luminescence temperature reading can be prospectively fed back into a temperature control loop to tune the RF heat supplied. Computational Fluid Dynamics (CFD) heat transport simulations supported the feasibility to establish finite and steady temperature differences between two particulate solid catalysts blended in a gas–solid reactor featuring unconventional RF energy input (see Section 1.3 in the ESI† for details). As summarized in Fig. 1d, the simulations predict a permanent temperature difference between the particles of the “hot” (magnetically heated) and “cold” catalysts, while the magnitude of the inter-catalyst temperature gap, averaged over the packed-bed, depends on various operational settings, *i.e.* radiofrequency energy input, reactor cooling, catalysts volume ratio, and gas space velocity, as well as the overall bed operation temperature range which may be modulated *e.g. via* both the RF frequency and power input as well as the T_Curie_ of the magnetic susception functionalities (Fig. S1 and S2 in the ESI†).

Experimentally, cobalt ferrite (CoFe_2_O_4_) nanocrystals were synthesized as magnetic susceptors. The material showed average crystalline domain and nanoparticle sizes of 26 nm and 25 nm, respectively, suggesting monocrystallinity (Fig. S3†). To avoid (unselective) contributions from this auxiliary functionality to catalysis, the surface of the nanosusceptors was blocked *via* coating with an overlay of non-porous, catalytically inert SiO_2_. Following coating, high-angle annular dark-field scanning-transmission electron microscopy (HAADF-STEM) coupled to energy dispersive X-ray spectroscopy (EDS) revealed assemblies of few CoFe_2_O_4_ nanocrystals, enveloped by a continuous SiO_2_ shell (Fig. S4†). The CoFe_2_O_4_@SiO_2_ susceptors showed a ferromagnetic behaviour with a saturation magnetization of 71 emu per g CoFe_2_O_4_ and a *T*_Curie_ of 703 K (Fig. 2a and b). The RF induction heating performance was evaluated in a packed-bed reactor, under conditions representative for catalysis conversion. As shown in Fig. 2c, on exposure to radiofrequencies (200 kHz), the solid reached temperatures between RT-623 K, modulable through the intensity of the magnetic field. Furthermore, even at the highest temperature level, it showed a remarkable stability to both steady (>70 h) and cyclic heating/cooling conditions (Fig. S5†), indicating the suitability of the CoFe_2_O_4_@SiO_2_ nanosusceptors to induce stable and swiftly cyclable catalysis-relevant temperatures by magnetic heating under industrially significant conditions. Additionally, two thermophosphor functionalities have been designed to show luminescence lines which can be simultaneously excited with a single-wavelength laser input, while luminescence emissions occur at sufficiently spaced wavelengths, thus enabling their separate detection to attain a material-specific thermometry. Y_2_O_3_ nanoparticles doubly doped with Tb and Eu were synthesized by a colloidal route (Fig. S6†). Following coating with a catalytically inert SiO_2_ shell and air-calcination, the material consisted of polycrystalline monodispersed Y_2_O_3_:Tb:Eu nanospheres (*d* = 298 ± 27 nm) wrapped in a continuous SiO_2_ shell (*t* = 41 ± 10 nm) (Fig. S6 and S7†). The non-porous nature of the SiO_2_ shell was corroborated *via* N_2_ physisorption analysis, which revealed identical and fairly low specific surface areas (4.5–4.8 m^2^ g^−1^) and pore volume values (<10^−2^ cm^3^ g^−1^) for both the uncoated and SiO_2_-coated Y_2_O_3_:Tb:Eu nanospheres (Fig. S8†). Cr-doped α-Al_2_O_3_ was also synthesized as a second thermophosphor (Fig. S9†). The photoemission properties of these materials were evaluated with transient laser photoluminescence spectroscopy (full details in the Supplementary Information).

**Fig. 2:**
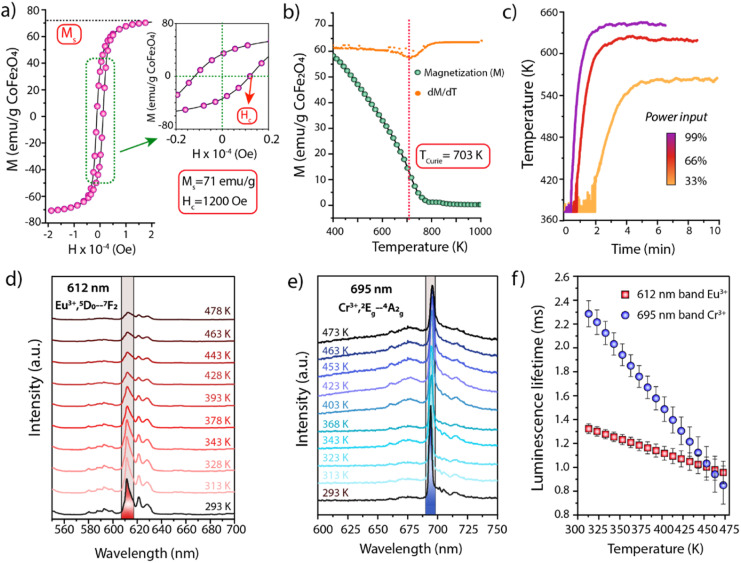
(a–c) Characterization of CoFe_2_O_4_@SiO_2_ magnetic nanosusceptors. (a) Magnetization (*M*) *vs.* applied field strength (*H*) curve for the determination of coercivity (*H*_c_) and saturation magnetization (*M*_s_). (b) Magnetization *vs.* temperature (*M*(*T*)) curve (green points) and the corresponding 1^st^ derivative (orange points). The Curie temperature corresponds to the inflection point of the *M*(*T*) trace. (c) Dynamic temperature response, as determined by remote wall pyrometry, of a packed-bed reactor filled with the CoFe_2_O_4_@SiO_2_ nanosusceptors upon modulation of the power output at the generator of a RF magnetic field (200 kHz) (see Experimental details in Section 1.2.8 of the ESI†). (d and e) Temperature-resolved luminescence emission spectra obtained upon excitation of Y_2_O_3_:Tb:Eu@SiO_2_ (d) and α-Al_2_O_3_:Cr (e) thermophosphors with a pulsed (<5 ns) 266 nm laser. (f) Luminescence lifetime *vs.* temperature calibration curves for Y_2_O_3_:Tb:Eu@SiO_2_ (emission band at 612 nm) and α-Al_2_O_3_:Cr (emission at 695 nm) thermophosphors. Error bars correspond to the standard error of the mean. A complete description of the luminescence lifetime calculation protocol can be found in Section 1.2.7 of the ESI.†

As shown in Fig. 2d and e, the Eu^3+^,^5^D_0_ → ^7^F_2_ and Cr^3+^,^2^E_g_ → ^4^A_2g_ luminescence modes in Y_2_O_3_:Tb:Eu and α-Al_2_O_3_:Cr phosphors can both be excited with a UV pulsed laser at a wavelength *λ* = 266 nm, while the luminescence emissions occur in the visible spectral range at *λ* = 612 and *λ* = 695 nm, respectively, *i.e.* sufficiently distant to be resolved and monitored simultaneously. Moreover, the corresponding luminescence lifetimes showed a significant temperature dependence in the range of 298–473 K in both cases, providing thermometry sensitivities of 2.3 and 9.1 μs K^−1^ for the Y_2_O_3_:Tb:Eu@SiO_2_ and α-Al_2_O_3_:Cr phosphors respectively (Fig. 2f).

To validate the concept of catalyst-specific heating and thermometry, we have selected a tandem process consisting of the integration of (i) ethene dimerization to 1-butene, followed by subsequent (ii) 1-butene isomerization and metathesis, with additional ethene, to produce propene in a single conversion step. This process holds potential in the context of *on purpose* propene production concepts, which are essential to bridge the so-called *propylene* demand/capacity *gap*.^16^ Additionally, a process which is functional at atmospheric pressure offers prospects for downstream catalytic upgrading of ethene-rich outlet streams from ethane crackers, avoiding costly intermediate gas pressurization steps.

Composite materials incorporating catalytic, as well as luminescence thermometry and magnetic susception auxiliary functionalities (the latter only for the *hot catalyst*) were assembled by physical integration of the individual nanosized components and conformed as consolidated particles. Regarding the catalytic functions, nickel dispersed on a mesostructured aluminosilicate (Ni/Al-SiO_2_) was selected as ethene dimerization (ED) catalyst, while Re supported on ultrastable Y zeolite (Re/USY) was chosen as catalyst for olefin metathesis (OM). For both materials, X-ray diffraction (XRD) and electron microscopy (Fig. S10 and S11†), complemented with X-ray absorption spectroscopy (XAS) (Fig. S12†), indicated the presence of highly dispersed metal species, lacking long-range atomic ordering. Next to Ni/Al-SiO_2_ (ED catalytic function), CoFe_2_O_4_@SiO_2_ (magnetic susception function) and Y_2_O_3:_Tb:Eu@SiO_2_ (luminescence thermometry function), nanocrystalline SiC was incorporated as high-thermal conductivity binder to minimize intraparticle temperature gradients in the *hot catalyst* composite. As shown in Fig. 3, cross-sectional EDS elemental maps for this multifunctional composite confirmed the integration of all individual components in mesoscale spatial proximity, as desired to ensure the steering of the catalytic function *via* localized magnetic heating, as well as the direct temperature probing *via* the thermophosphors. Similarly, Re/USY and α-Al_2_O_3_:Cr were incorporated as OM catalytic and thermometry functions, respectively, in the *cold catalyst* composite. While Ni- and Re-based solid catalysts are selective catalysts for ED and OM reactions,^17^ respectively, they typically require different reaction temperatures for optimal operation. This is illustrated in Fig. 4a and b, which summarizes the performance of the Ni/Al-SiO_2_ ED and Re/USY OM catalysts, individually in the temperature range of 323–453 K, under conventional, *i.e.* isothermal packed-bed operation. The ethene dimerization rate on Ni/Al-SiO_2_ increased monotonically with temperature over the entire temperature range, while the selectivity to butenes (C_4_^=^) was invariably high (>85%) at all temperatures (Fig. 4a). Hence, the highest temperature is preferred to maximize the yield to butenes in this case.

**Fig. 3:**
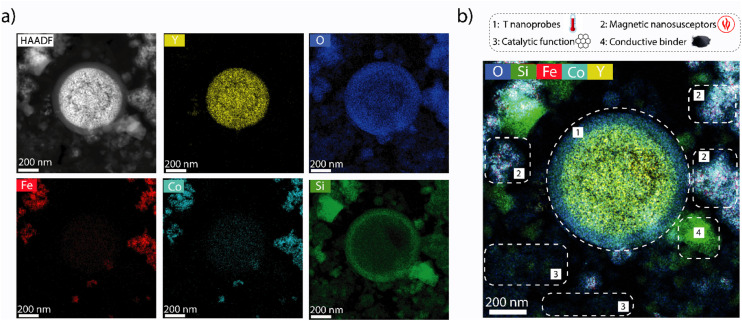
Nanospatial arrangement of functionalities in a multifunctional composite catalyst. (a) Cross-sectional HAADF-STEM micrograph and the corresponding EDS compositional maps for the *hot catalyst* composite material, incorporating Ni/Al-SiO_2_ (ethene dimerization catalyst function), CoFe_2_O_4_@SiO_2_ (magnetic susception function), Y_2_O_3_:Tb:Eu@SiO_2_ (luminescence thermometry function) and nanosized SiC (thermally conductive binder) in a 45/25/15/15 wt ratio. (b) Combined EDS compositional map with representative chemical elements from each component nanomaterial. The areas delimited by the dashed lines identify the different components as derived from the local compositions (see legend).

**Fig. 4:**
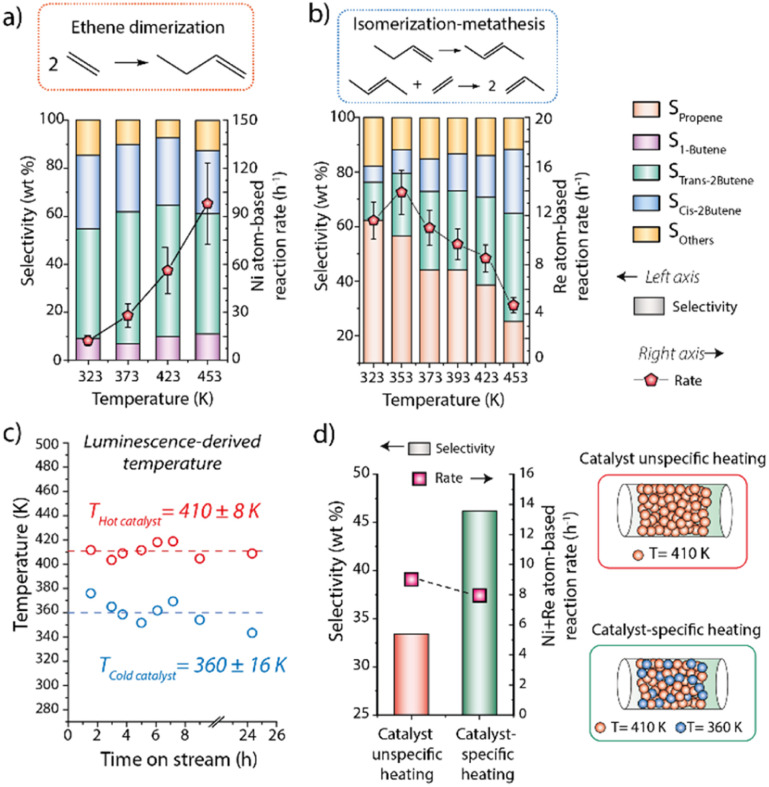
Metal-specific conversion rate and selectivity for (a) ethene dimerization on Ni/Al-SiO_2_; and (b) 1-butene/ethene isomerization/metathesis on Re/USY as a function of the reaction temperature in the range of 323–453 K (*P* = 1 bar). (c) Evolution of the catalyst-specific temperature for *hot* and *cold* catalysts, as determined by *operando* luminescence thermometry, with time-on-stream under reaction conditions for the tandem isomerization/metathesis conversion of ethene to propene. The *x*-axis includes a break between 10 and 22 hours on stream due to the need to halt the high-power laser, not the catalytic reaction, when the centralized laser cooling system was not operative overnight. The luminescence-based temperature reading was resumed afterwards, following 24 h on-stream, proving the stability of the catalyst-specific temperatures. Uncertainty values indicated for the temperatures on the plot correspond to the maximum deviation observed for the entire set of data with respect to the average value. (d) Comparison of the catalytic performance in the tandem dimerization/isomerization/metathesis conversion of ethene to propene under conventional catalyst-unspecific (convective) heating and catalyst-specific (magnetic) heating, respectively. Performance values correspond to 18 h time-on-stream.

In marked contrast, olefin metathesis catalyzed by Re/USY showed a maximum reaction rate at 353 K, while at higher temperatures significant catalyst deactivation sets in (see Fig. S13† for an analysis of the deactivation kinetics), leading to progressively lower metathesis rates on increasing temperature (Fig. 4b). Moreover, the selectivity to propene sank noticeably too on increasing temperature, confirming that operation temperatures ≥373 K are undesired for optimal performance in this case. These results emphasize the different optimal operation temperature for each individual reaction.

Next, the concept of catalyst-specific heating and thermometry was explored through the application of the multifunctional composite catalysts. Particles of both composite materials were blended in the packed-bed reactor, and the mixed catalyst bed was applied to convert an ethene feed stream under RF energy input. *Operando* luminescence thermometry was applied to simultaneously monitor the temperature of the particles of each catalyst during reaction. With feedback from catalyst-specific thermometry, process conditions were adjusted to attain a temperature reading of *ca.* 410 K for the *hot* (ED) *catalys*t. Under these conditions, the average temperature registered for the *cold* (OM) *catalyst* was 360 K. Moreover, this 50 K inter-catalyst temperature difference could be steadily sustained under operation conditions for at least 24 hours (Fig. 4c). Under these conditions, an overall ethene conversion rate of 8.0 h^−1^ was attained, alongside a selectivity to propene of 46% after 18 h of operation (Fig. 4d). For comparison, a test was performed under conventional *i.e.* catalyst-unspecific heating, resulting in an isothermal bed at 410 K. An essentially identical ethene conversion rate of 8.9 h^−1^ was obtained in this case, as expected given that the average temperature of the ED catalyst is the same in both tests. However, the selectivity to propene was noticeably lower (33%), becoming this difference more significant at longer time-on-stream (Fig. S14†). These results evidence the detrimental effects of imposing a common, higher reaction temperature for the OM catalyst and underscoring the benefits of decoupling the average operation temperatures for each of the integrated catalysts.

In summary, a multifunctional material design has been combined to unconventional energy input by magnetic heating, and contactless luminescence thermometry, to introduce the concept of catalyst-specific heating and thermometry in tandem catalysis. Feedback on the working temperature under *operando* conditions, and with catalyst specificity, allows to establish, assess, and ultimately adjust a steady temperature difference between two proximate solid catalysts. The new concept provides means to circumvent temperature incompatibilities which undermine, or directly rule out, several conceivable tandem catalytic conversions, as well as other processes where the single-pot cooperation of different functionalities, *e.g.* catalysis, sensing, molecular sieving, *etc* with different optimal operation temperatures, is desired.

## Conflicts of interest

There are no conflicts to declare.

## Supplementary Material

TA-011-D3TA03654E-s001
