# Supplementary material for: Multifunctional materials for catalyst-specific heating and thermometry in tandem catalysis
Source: J Mater Chem A Mater. 2023 Sep 13;11(37):19854–9. doi: 10.1039/d3ta03654e (PMC10521348; doi:10.1039/d3ta03654e)
Supplement: TA-011-D3TA03654E-s001 [file TA-011-D3TA03654E-s001.pdf]

Electronic Supplementary Material (ESI) for Journal of Materials Chemistry A.
This journal is © The Royal Society of Chemistry 2023

Supplementary information

Multifunctional materials for catalyst-specific heating and thermometry in tandem catalysis

Marcos G. Farpón[a,b], Raquel Peláez[a], Verónica Recio[a], Burak Atakan[c], Carlos Zaldo[d], Gonzalo Prieto[a,b,*]

[a] ITQ Instituto de Tecnología Química, Universitat Politècnica de València-Consejo Superior de Investigaciones Científicas (UPV-CSIC), Avenida de los Naranjos s/n, Valencia 46022, Spain.

[b] Max-Planck-Institut für Kohlenforschung, Kaiser-Wilhelm-Platz 1, 45470 Mülheim an der Ruhr, Germany.

[c] Thermodynamik ,EMPI, Faculty for Engineering, University of Duisburg-Essen, Lotharstr. 1, 47057, Duisburg, Germany

[d] Instituto de Ciencia de Materiales de Madrid, Consejo Superior de Investigaciones Científicas CSIC, c/ Sor Juana Inés de la Cruz 3, 28049 Madrid, Spain

* Correspondence to G. Prieto: prieto@itq.upv.es

Table of contents

1. EXPERIMENTAL METHODS 4

1.1. Material synthesis 4

1.1.1. Catalytic functional components 4

1.1.1.1. Ni/Al-SiO_2 ethene dimerization catalyst function 4

1.1.1.2. Re/USY ethene dimerization catalyst function 4

1.1.2. Auxiliary functional components 5

1.1.2.1. $CoFe_2O_4$ nanosusceptor function 5

1.1.2.2. Y_2O_3:Tb:Eu thermometric function 5

1.1.2.3. α-Al_2O_3:Cr thermometric function 6

1.1.3. Multifunctional catalyst assembly 6

1.1.3.1. Multifunctional “hot” ethene dimerization catalyst 7

1.1.3.2. Multifunctional “cold” olefin metathesis catalyst 7

1.2. Characterization methods 7

1.2.1. Inductively-coupled plasma optical-emission spectroscopy (ICP-OES) 7

1.2.2. X-Ray diffraction (XRD) 7

1.2.3. (Scanning-) Transmission Electron Microscopy ((S)TEM) 8

1.2.4. N_2 physisorption 8

1.2.5. X-Ray Absorption Spectroscopy (XAS) 8

1.2.6. Magnetization measurements 9

1.2.7. (Operando) luminescence thermometry 9

Figure EM1. 11

1.2.8. Heating power assessment of magnetic susceptors 12

1.3. CFD heat transfer simulations 12

1.3.1. Geometry, model and boundary conditions 13

Table EM1 14

Figure EM2. 15

1.3.2. Properties and operational parameters considered. 15

1.3.3. Sensitivity studies and average temperature gradient determination 15
Table EM2 16
1.4. Catalytic conversion experiments 16
Figure EM3. 20
Figure EM4 21
2. SUPPORTING FIGURES 22
Figure S1. 22
Figure S2: 24
Figure S3: 25
Figure S4 26
Figure S5: 27
Figure S6: 28
Figure S7: 29
Figure S8: 30
Figure S9 31
Figure S10 32
Figure S11 33
Figure S12 34
Figure S13 35
Figure S14 36
3. SUPPORTING TABLES 37
4. REFERENCES 38

1. EXPERIMENTAL METHODS

1.1. Material synthesis

1.1.1. Catalytic functional components

1.1.1.1. Ni/Al-SiO_2 ethene dimerization catalyst function

A mesostructured aluminosilicate Al-MCM-41 (Al-SiO_2) was synthesized via hydrothermal synthesis adapting a previously reported method.[1] In a typical synthesis, 13.67 g of hexadecyltrimethylammonium bromide ($C_{19}H_{42}BrN$ or $C_{16}TABr$, Thermo Scientific >99 %) and 23.86 g of tetramethylammonium hydroxide ($C_4H_{12}NOH$ or TMAOH, Sigma Aldrich, 25 wt% in water) were dissolved in 91.6 g of mili-Q water and magnetically stirred in a water bath, preheated at 373 K, for 1 h. Subsequently, 0.488 g of aluminum hydroxide (Wako, 95% purity) were dissolved and the solution was stirred for 30 min. Afterwards, the solution was transferred into a polypropene bottle containing 15 g of colloidal high-purity SiO_2 (Aerosil 200, Evonik) and the resulting mixture was mechanically stirred with an overhead laboratory stirrer at 120 rpm for 2 hours. Afterwards, an adequate amount of milli-Q water was added to reach a synthesis gel molar composition of 1 SiO_2: 0.15 $C_{16}TABr$: 0.26 TMAOH: 0.0125 Al_2O_3: 24.3 H_2O. The gel was transferred into a PTFE-lined stainless-steel autoclave and heated in an oven at 403 K for 44 hours under static conditions. The product was recovered by filtration, washed with 2 L of deionized water preheated at 373 K, and dried in air at 373 K for 6 hours. To remove the porogen agent, the dry solid was transferred into a tubular quartz reactor in the form of a packed bed, and heated in an vertically oriented tubular furnace to 813 K with a heating rate of 3 K min^{-1} from RT, under top-down flow (200 mL min^{-1}) of firstly N_2 (during the heating ramp and 1 h at 813 K) and then synthetic air for 6 h at 813 K. Ni/Al-SiO_2 was synthesized via ion exchange of the previously synthesized Al-SiO_2 with a Ni^{2+} precursor salt. 10 g of Al-SiO_2 were suspended in 100 mL of deionized water and the suspension was sonicated for 15 minutes. Afterwards, 12.58 g of $Ni(NO_3)_3 \cdot 6H_2O$ were dissolved in 60 mL of deionized water and added dropwise over the Al-SiO_2-contaning suspension under magnetic stirring. The solid was recovered by filtration and washed with 4 L of deionized water and 500 mL of acetone, and finally dried in air at 373 K.

1.1.1.2. Re/USY ethene dimerization catalyst function

Re/USY was synthesized by a wet impregnation approach.[2] Firstly, commercial NH_4-USY (Ultra Stable Y) zeolite with a Si/Al molar ratio of 6 (Zeolyst, CBV 712) was calcined under synthetic air flow in a tubular packed-bed quartz reactor at 823 K for 4 hours with a heating rate of 3 K min^{-1} from RT to obtain the zeolite in its protonic form (H-USY). Afterwards, 0.305 g of NH_4ReO_4 (Sigma Aldrich, >99 % purity) were dissolved in 20 mL deionized water and stirred at room temperature for 15 minutes. 5 g of H-USY were suspended into the previous solution and stirred for 1 hour. The

solution was dried in an oven at 353 K, under static conditions, overnight and the resulting powder was calcined in a tubular packed-bed quart reactor at 823 K for 4 hours with a heating rate of 1 K min^{-1} from RT under top-down synthetic air flow (200 mL min^{-1}).

1.1.2. Auxiliary functional components

1.1.2.1. $CoFe_2O_4$ nanosusceptor function

Cobalt ferrite ($CoFe_2O_4$) nanocrystals were synthesized by coprecipitation.[3] In a typical synthesis, 1.9161 g of $Co(NO_3)_2·6H_2O$ (Sigma Aldrich, >98 % purity) and 5.3028 g of $Fe(NO_3)_3·9H_2O$ (Sigma Aldrich, >98 %) were dissolved in 26 mL of deionized water and magnetically stirred for 15 minutes. In parallel, a second solution of sodium hydroxide was prepared by dissolving 2.57 g of NaOH (Sigma Aldrich, 97% purity) in 65 mL of deionized water. This latter solution was added at a constant rate of 5 mL min^{-1} over the metals-containing solution at 353 K. After the complete addition of the sodium hydroxide solution, the mixture was magnetically stirred at 353 K for 2 hours. Next, 7 droplets of oleic acid (Sigma Aldrich,>99 %) were added as capping agent. After 15 minutes of homogenization, the solid product was recovered via ultracentrifugation (6000 rpm), washed with ethanol and deionized water, dried at 373 K and eventually calcined in a muffle oven under stagnant air at 973 K for 4 hours with a heating rate of 3 K min^{-1} from RT.

The cobalt ferrite nanoparticles were coated with a non-porous silica shell ($CoFe_2O_4@SiO_2$) following an adapted Stöber method.[4] Firstly, 0.5 g of $CoFe_2O_4$ synthesized as described in the previous step were suspended in a mixture of 91 mL of deionized water and 477 mL of absolute ethanol. The suspension was magnetically stirred at room temperature for 15 minutes and then ultrasonicated in an ultrasonicator bath (Branson 3800, 110 W) for another 15 minutes. Afterwards, 8.85 mL of an ammonium hydroxide solution (Panreac, 25 % NH_3 in water,) were added and the suspension stirred for another 10 minutes. Finally, 8.85 mL of tetraethyl orthosilicate (TEOS, Sigma Aldrich >99 %) were added at once under stirring, and the suspension was kept stirring at room temperature overnight to complete TEOS hydrolysis and condensation. The solid was recovered by ultracentrifugation (6000 rpm), washed with ethanol and water, dried in air at 373 K and finally calcined at 973 K for 4 hours in a muffle oven under stagnant air atmosphere using a heating rate of 3K min^{-1} from RT.

1.1.2.2. Y_2O_3:Tb:Eu thermometric function

Yttrium oxide nanoparticles doped with europium and terbium (Y_2O_3:Tb:Eu) were synthesized by following an urea-mediated co-precipitation route.[5] In a synthesis batch, 6.28 g of $Y(NO_3)_3·6H_2O$ (Sigma Aldrich, 99.8%), 0.193 g of $Eu(NO_3)_3·6H_2O$ (Alfa Aesar, 100%) and 0.188 g

of $Tb(NO_3)_3 \cdot 6H_2O$ (Sigma Aldrich, 99.9%) were co-dissolved in 1.2 L of a 0.5 M urea (Sigma Aldrich, 99.5%) aqueous solution. The molar ratio of nitrate precursors was set to attain nominal lanthanide loadings of 2.5 at% Eu, and 2.5 at% Tb respect to the total metal content, respectively, in the Y_2O_3 matrix. The solution was stirred until total homogenization at room temperature in a jacketed glass reactor. Afterwards, the solution was heated to 363 K within one hour using a thermostated bath to feed the jacket of the glass reactor and kept at this temperature for another 2 hours. Then the solution was let cooled down to 323 K and the solid products were recovered by ultracentrifugation (9000 rpm) and then washed three times with deionized water and once with ethanol.

Next, the Y_2O_3Tb:Eu nanoparticles were coated with a non-porous silica shell (Y_2O_3:Tb:Eu @SiO_2) by using a modified Stöber method similar to the one described above for the coating of cobalt ferrite nanocrystals. In a standard procedure, 2 g of Y_2O_3:Tb:Eu were mixed in 83.47 g of absolute ethanol and 20.03 g of deionized water, stirred for 10 minutes until homogenization, and ultrasonicated an ultrasonicator bath (Branson 3800, 110 W) for another 10 minutes. Afterwards, 1.94 mL ammonium hydroxide (28-30 % NH_3 in water, Panreac 25 %) were added and the suspension stirred for 10 minutes. Finally, 3.86 mL of tetraethyl orthosilicate (TEOS, Sigma Aldrich >99 %) were added at once under stirring, and the solution was kept stirring at room temperature overnight to complete TEOS hydrolysis and condensation. The solid was then recovered by ultracentrifugation (9000 rpm), washed with water and ethanol, and finally dried in air at 353 K. The resulting material was calcined under stagnant air atmosphere in a muffle oven at 973 K for 4 hours with a heating rate of 3 K min^{-1}.

1.1.2.3. α-Al_2O_3:Cr thermometric function

The α-Al_2O_3:Cr thermographic phosphor was synthesized by mixing 0.78 g of $Cr(NO_3)_3 \cdot 9H_2O$ (Sigma Aldrich, 99% purity) and 15.59 g of pseudo-böhmite (AlO(OH), Disperal P2, Sasol) in a mortar and grinding with a pestle until obtaining an homogeneous blue-greyish powder. Afterwards, the material was calcined and crystallized in a muffle oven under stagnant air atmosphere at 1573 K for 6h with a heating rate of 3 K min^{-1} from RT.

1.1.3. Multifunctional catalyst assembly

Multifunctional *composite* catalysts were confirmed by co-grinding of the different nanosized components followed by pelletizing and pellet crushing.

1.1.3.1. Multifunctional "hot" ethene dimerization catalyst

Nanocrystalline SiC (nc-SiC). was incorporated as a binder into the composite material to improve the overall thermal conductivity and minimize intraparticle temperature gradients. Prior to its use, and to remove potential contaminants, commercial SiC nanopowder was calcined in a muffle furnace under stagnant air atmosphere at 623 K for 4 h with a heating rate of 3 K min^{-1} from RT. Adequate amounts of Ni/Al-SiO_2, $CoFe_2O_4$@SiO_2, Y_2O_3:Tb:Eu@SiO_2 and nc-SiC, to reach a nominal composition of 45/25/15/15 (wt/wt), were mixed and gently co-ground in a mortar until reaching an homogeneous dark-grey powder. Afterwards, the powder was pressed using a stainless-steel die (32 mm diameter) and a Specac press, applying a pressure of 5 tons (770 bar). The pellet was then crushed and ground again, and the whole protocol was repeated several times until complete homogenization.

1.1.3.2. Multifunctional "cold" olefin metathesis catalyst

Adequate amounts of Re/USY and α-Al_2O_3:Cr, to reach a nominal composition of 85/15 (wt/wt), were mixed and gently co-ground in a mortar until reaching an homogeneous light pink powder. Analogous pressing and crushing protocol as the one described in section 1.1.3.1 was followed.

1.2. Characterization methods

1.2.1. Inductively-coupled plasma optical-emission spectroscopy (ICP-OES)

Bulk material compositions were determined by inductively-couple plasma optical-emission spectrometry (ICP-OES) in a Thermo Scientific iCAP PRO spectrometer, previously calibrated using commercial standard solutions. The solid samples were disaggregated in *aqua regia* (3:1 v:v HCl:HNO_3) prior to injection into the nebulizer of the spectrometer.

1.2.2. X-Ray diffraction (XRD)

Powder XRD Measurements were performed in Bragg-Brentano geometry using a PANalytical CUBIX diffractometer equipped with a PANalytical X'Celerator detector. X-ray radiation of Cu Kα (λ_1 = 1.5406 Å, λ_2 = 1.5444 Å, I_2/I_1 = 0.5) was used, operating the X-ray sourcing tube at a voltage and intensity of 45 kV and 40 mA, respectively. The length of the goniometer arm was 200 mm, and a fixed divergence slit with a 1/8˚ aperture was used. The measurement range was from 3.5˚ to 90.0˚ (2θ), with a step of 0.020˚ (2θ) and a measurement time of 35 seconds per step. Low angle XR patterns were collected using instead a fixed divergence slit with a 1/32˚ aperture. In

this case, the measurement range was from 0.65° to 7° (2θ), with a step of 0.020° (2θ) and a measurement time of 20 seconds per step. In all cases, the measurements were carried out at 298 K, rotating the sample at 0.5 revolutions per second.

1.2.3. (Scanning-) Transmission Electron Microscopy ((S)TEM)

(High-resolution-) Transmission Electron microscopy (HR-TEM) and High-Angle Annular Dark-Field Scanning-Transmission electron microcopy (HAADF-STEM) experiments were carried out either in a TITAN G2 microscope (Thermo Fisher, formerly FEI) equipped with a Schottky-type Field-Emission Gun and operated at an acceleration voltage of 300 kV, or in a JEOL JEM-1400 Flash operated at an acceleration voltage of 120 kV. Prior to observation, the samples were embedded in a low-viscosity epoxy resin (Spurr). Then, slices of the embedded material were produced, with nominal thickness of 200 nm, on a Reichert Ultracut ultramicrotome mounting a Diatome diamond knife and deposited onto Cu TEM grids (150 mesh) coated with a continuous formvar film. Cross-sectional compositional maps were generated from the ultramicrotomed specimens by means of a Super X quadruple Energy-Dispersive Spectroscopy (EDS) detector with automated drift correction. Particle and shell size distributions were determined by assessing at least 100 items on the calibrated micrographs, reporting the average value and the standard deviation values, respectively.

1.2.4. N_2 physisorption

Nitrogen adsorption isotherms were registered using an ASAP 2420 apparatus (Micromeritics) at −196 °C (77 K). Before the analysis, around 200 mg of the sample (sieve fraction 0.2-0.4 mm) were degassed at 673 K and ~5×10^{-6} bar overnight. The specific surface area was determined by applying the Brunauer−Emmett−Teller (BET) equation in the relative pressure range (P/P^o) of 0.05-0.3. Pore size distributions and total pore volume were determined using the Barret-Joyner-Halenda (BJH) method for the desorption branch datapoints. Micropore volume was determined using the t-plot method, whereas mesopore volume was determined by subtracting the micropore volume from the total pore volume value.

1.2.5. X-Ray Absorption Spectroscopy (XAS)

X-ray absorption spectra were recorded at the Ni K-edge (8.333 keV) and Re-L3 edge (10.535 keV) at the CLÆSS beamline (BL22) of the ALBA synchrotron light source (Spain). The beam was monochromatized using a (311) Si double crystal monochromator and harmonic rejection was performed using a Pt-coated silicon mirror. Reference Ni foil and high-purity compounds, i.e.

NiO (Sigma Aldrich, 99.8%), Re_2O_7 (Sigma Aldrich, >99.9 %) and Re powder (ABCR99.9 %), were pressed into self-supported pellets (Ø=13 mm), with optimized thickness after dilution in powder boron nitride, and measured in transmission mode employing ion chambers filled with appropriate gases in order to adsorb 15% and 80% of the photons at I_0 and I_1 ion chambers, respectively. For Ni-based and Re-based catalysts, Extended X-ray Absorption Fine-Structure (EXAFS) spectra were acquired at room temperature in transmission mode using a multi-channel Silicon Drift Detector. XAS data reduction and extraction of the χ(k) function has been performed using the Athena code from the Demeter software package (version 0.9.26).[6] EXAFS data were analyzed in FEFF6 code. In the data reduction process, a k-range up to 12 $Å^{-1}$ was considered. In all cases, a Rbkg value of 1 was considered.

1.2.6. Magnetization measurements

For nanosized magnetic susceptors, the magnetization (M) was measured as a function of the applied magnetic field (H) at room temperature. M(H) curves were measured at room temperature (RT) by means of a vibrating sample magnetometer (VSM) Microsense(r) EV9 in the field range +/- 20 kOe. Subsequently, M(T) curves were recorded under 1 kOe and 10kOe from 300 K to 1000 K with a heating rate of 10 K min^{-1} under argon atmosphere. About 20 mg of the powder sample were packed into a 3 mm-diameter quartz cup, which was attached to a rod that transmits the vibration from the linear motor by using 901 alumina adhesive ceramic. The Curie temperature (T_{Curie}) was determined by finding the temperature for the minimum of the 1st derivative function *d*M(T)/*d*T, corresponding to the inflection point in the decaying M(T) trace.

1.2.7. (Operando) luminescence thermometry

For the luminescence thermometry measurements, Y_2O_3:Tb:Eu@SiO_2 and α-Al_2O_3:Cr phosphors were remotely excited by the third harmonic (λ=266 nm) of a Spectra Physics Nd-YAG laser (model Quanta Ray), providing pulses with duration <5 ns at a repetition rate of 10 Hz. To avoid residual contributions from the fundamental (λ=1064 nm) and other harmonics (λ=532 and 355 nm) laser emissions the laser output was filtered by a series of two dichroic mirrors (highly reflecting at 266 nm) and a bandpass filter (Semrock FF01-260/15-25).

First, the fluorescence emission spectra of the phosphors were recorded by using an optical fiber (100 μm of core diameter) coupled compact Spectral Products spectrometer (model SM440). The fluorescence was focused onto the fiber core by a set of BK7 lenses. The spectrometer incorporates a Toshiba CCD detector with 3648 pixels which covers the range λ= 200 to 1050 nm. In the short wavelength end the spectral detection is limited to 250 nm by the transmission of the

fiber. The entrance slit width of the spectrometer is 50 micrometers what sets the spectral resolution in between 0.2 nm (for 200 nm light) and 0.5 nm (for 1050 nm light).

Next, fluorescence lifetime measurements were made for different probe temperatures at the wavelength corresponding to maximum emission intensity for each probe: λ_{EMI}= 613 nm for Eu^{3+} and λ_{EMI} = 694 nm for Cr^{3+}, respectively. In this case the thermometric probe luminescence was collected by a set of BK7 lenses, dispersed by a SPEX (f=34) Czerny-Turnet monochromator and detected by a Peltier cooled photomultiplier tube (model R2658). The time dependent luminescence intensity traces were stored and averaged (typically 10^3 acquisitions) in a 1GHz digital Tektronics oscilloscope. As expected from the presence of energy transfer processes and multiphonon emission losses, the decay profiles were not single exponential. To circumvent these issues, an effective luminescence lifetime was calculated according to equation 1.

$$\tau = \frac{\int_0^t I(t)x\, tdt}{\int_0^t I(t)} \qquad \text{Eq.1}$$

wherein τ stands for the effective luminescence lifetime, t stands for time and $I(t)$ corresponds to the emission intensity at a certain time.

Prior to *operando* catalyst-specific thermometry experiments, the thermal response of each of the respective thermophosphor probes (Y_2O_3:Tb:Eu@SiO_2 and α-Al_2O_3:Cr) was calibrated by using a horizontal furnace plate heated by a resistance, depositing the powdered probe material on a platinum chuck directly on top of the furnace. The powdered probe material was deposited on a platinum chuck directly on top of the resistor. Five K-type thermocouples buried into the powder at the center and at the edges of the irradiated area were used to assess possible thermal gradients during calibration. These thermocouples were simultaneously read over time by a TC Direct digital recorder (model TCD7000). The system was enclosed in a box to minimize temperature fluctuations associated to air convection. The nominal temperature was evaluated as the average of the five thermocouple readings, each averaged over time once the temperature fluctuations of the phosphor materials was lower than 5% of the reading for at least 10 min to ensure steady state. Calibrations were performed in the temperature range of 298-473 K, with 10-15 K intervals, and once the phosphor materials had been exposed to each temperature for at least 10 min to ensure steady state.

Phosphor thermometry calibrations were also performed for a mixture of the two sets of thermophosphors loaded into the cylindrical, quartz fixed-bed reactor applied for tandem catalysis

and *operando* catalyst-specific thermometry experiments (see section 1.4 in this Supporting Information for details on the reactor setup). The reactor was heated by means of a heat tracing element connected to an external PID temperature controller (Ascon Technologies, K48 Programmable). Calibrations were performed both prior to and following exposure to tandem catalysis conditions, in order to assess potential spectral overlapping phenomena and gas quenching effects due to the exposure to the reactive gas atmosphere. Alignment and calibration of the optics for the reactor geometry was checked via analysis of the lifetimes at room temperature, which gave identical values as those obtained with the horizontal oven during the separate calibration experiments. Figure EM1 shows an overview of the experimental setup. Results confirmed the absence of significant deactivation in the thermometry responses due to the exposure to reaction conditions. Hence, the luminescence lifetime-temperature calibration curves considered for *operando* thermometry were based on the average of the three experiments described above (see Figure 2f in the main text).

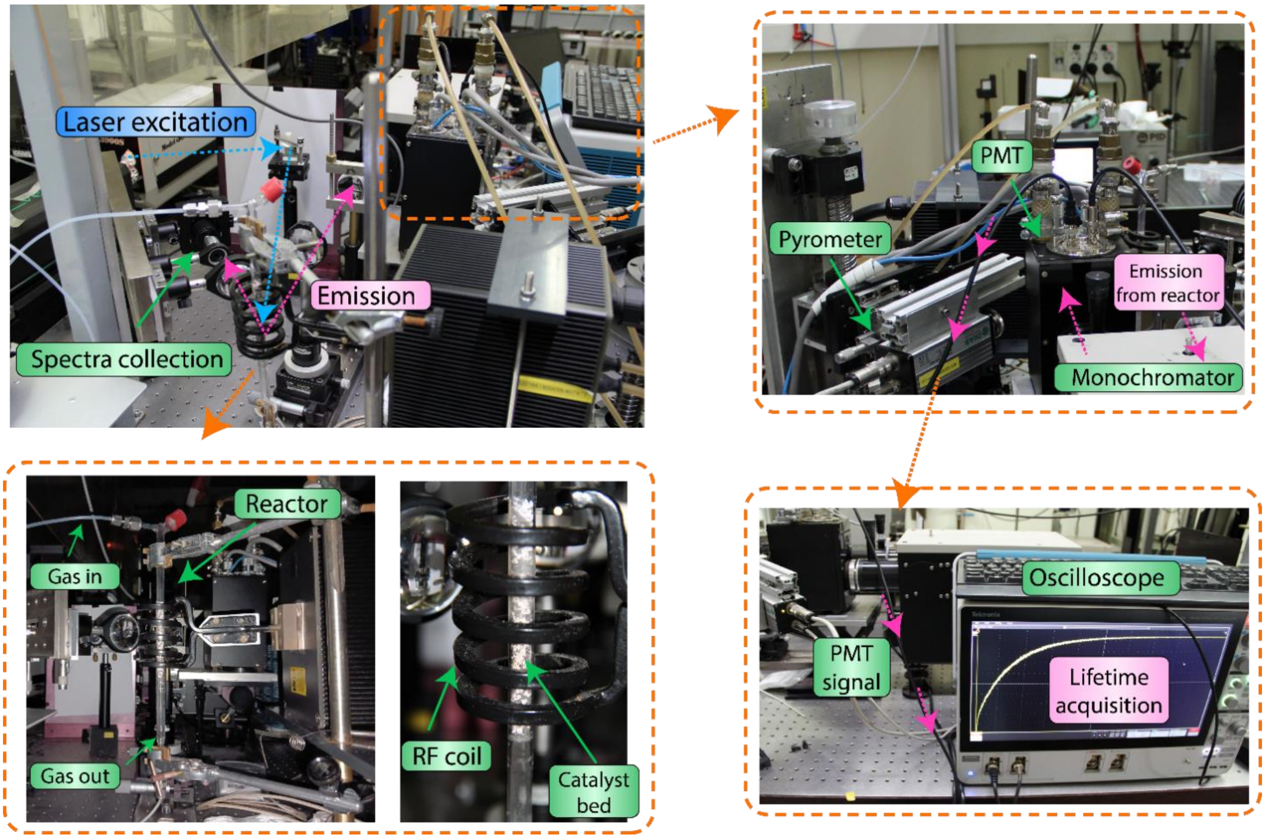

Figure EM1: Schematic overview of the experimental setup used for the *operando* catalytic experiments using the catalyst-specific heating and thermometry concept.

Uncertainty bars for luminescence lifetimes have been determined as the standard error of the mean (eq 2).

$$\sigma_{\bar{X}} = \frac{\sigma}{\sqrt{n}} \quad \text{Eq.2}$$

wherein $\sigma_{\bar{X}}$ is the standard error of the mean, σ is the standard deviation of the sample and n the number of independent experimental measurements as described above. The absolute sensitivity was defined as the absolute value of the first derivative of the lifetime with respect to temperature (eq 3),[7,8] which corresponds to the slope of the lifetime vs temperature calibration lines shown in Figure 2f (main manuscript).

$$S_a = \left|\frac{d\tau}{dT}\right| \quad \text{Eq.3}$$

1.2.8. Heating power assessment of magnetic susceptors

The heating capacity of $CoFe_2O_4@SiO_2$ under a RF field was evaluated by loading around 1 g of this material in a tubular quartz reactor (OD =20 mm, height 300 mm), containing a quartz sheath (OD=3 mm). Temperature evolution was monitored by using a pyrometer with a time resolution of 15 ms (see section 1.4 in this Supporting Information for full details on the reactor setup used and the pyrometer calibration details). The power of the RF generator (200 kHz) was tuned in the range 33-99% to modulate the intensity of the magnetic field and thus the heating capacity of the magnetic susceptors. For a selected experiment, heating-cooling cycles were performed by switching the RF generator (at a power output of 99 %) for a period of 30 minutes and then shutting it down for 10 minutes (considered this period as one cycle). This process was repeated for 3 cycles, while constantly monitoring the temperature in the packed bed. In another experiment, the temperature reached by the solid exposed to a steady RF energy input (99 % power output) was measured for ca. 72 h on stream to assess longer-term stability of the nanosized susceptors. In all cases, experiments were performed under a N2 flowrate of 15 mLN min^{-1} (see section 1.4 for the experimental details)

1.3. CFD heat transfer simulations

Computational Fluid Dynamics (CFD) simulations have been applied to assess the feasibility and the extent of establishing a steady and finite temperature difference between two particulate catalysts in a single packed-bed reactor through the catalyst specific heating and thermometry approach pursued in this study. Temperature profiles for a reactor model have been determined by

solving the momentum and energy equations of change using a finite elements method as implemented in Comsol Multhiphysics (version 5.5).

1.3.1. Geometry, model and boundary conditions

The reactor has been modelled with a discrete model, in which the solid particles are considered one by one to assess more precisely interparticle thermal gradients. The geometry selected consists of a cylindrical quartz tube (internal diameter= 5 mm and 15 mm length) containing a packed bed of spherical particles of 1 mm. To reduce the computational demand, a 2D model (i.e., considering symmetry in the angular or ϴ coordinate) has been considered, wherein the cross-sections of 75 spherical particles were randomly distributed along the packed-bed to attain an overall bed porosity (void fraction) of 0.36 (see Figure EM2). In addition, the maximum size of the particles has been set to 0.99 mm, which corresponds to 99% of the actual particle size. The latter strategy has been previously reported in the literature to circumvent meshing issues associated to interparticle contact points.[9,10]

The simulation has been performed by solving the continuity, Navier-Stokes and energy balance equations respectively (Eq 4-6), setting adequate boundary conditions (see table EM1). For the solution of the momentum equation, gas inlet, wall condition and outlet have been assigned to the boundaries corresponding to the reactor inlet, walls and outlet respectively (regions 1, 2 and 3 in Figure EM2). The wall condition has been also assigned to the external surface of all-solid particles. For the solution of the energy balance, the gas inlet temperature, heat removal via exchange across the reactor's wall to a heat transfer fluid (HTF), and heat outflow have been set in regions 1, 2 and 3 respectively. Regarding the boundary conditions for the *hot catalyst* particles (boundary 4), two different scenarios have been considered: (i) assuming the *hot catalyst* operates isothermally, i.e. mimicking the case wherein the magnetic susceptors self-regulate at their Curie temperature, and (ii) setting the *hot catalyst* as a heat source with a power proportional to the RF field intensity (hereafter referred to as CT and HS scenarios for (i) and (ii) respectively).

$$\nabla\,(\rho u) = 0 \qquad \text{Eq.4}$$

$$\rho\,(u\,.\nabla)\mathrm{u} = \nabla(-\mathrm{P\,I} + \tau\,) + \mathrm{F} \qquad \text{Eq.5}$$

$$\rho\, C_p u\,\nabla\mathrm{T} = \nabla(\mathrm{k}\,\nabla\mathrm{T}) \qquad \text{Eq.6}$$

wherein "ρ" and "u" correspond to the fluid density and velocity respectively, P to the modulus of the absolute pressure, I to the unit vector, "F" to external forces such as gravity, "k" to the solid thermal conductivity, "C_p" to the gas specific heat capacity at constant pressure and "T" to temperature. In all cases, the term "∇" stands for the gradient operator, which corresponds to the partial derivative of the dependent variable "y_n" respect to the independent variable "x_n".

Table EM1. *Boundary conditions set in the simulation model for the solution of the different case studies.*

Boundary	Type of condition	Equation
1	Inlet velocity	$u = u_{gas\ in}$
1	Temperature	$T = T_{gas\ in}$
2	Heat flux	$q = h\,(T - T_{coolant})$
3	Outflow	$-k\,(\nabla T) = 0$
4	Temperature	$T = T_{Hot\ catalyst}$
Solid particles and boundary 2	Wall condition	$u = 0$

The meshing was created with a tetrahedral-shaped geometry, setting the maximum element size to a value of 3.00×10^{-5} m to describe as much as possible the voids between the particles. Mesh convergence tests showed no significant deviations in the results when the element size was further reduced below the selected size, proving the validity of the selected meshing size.

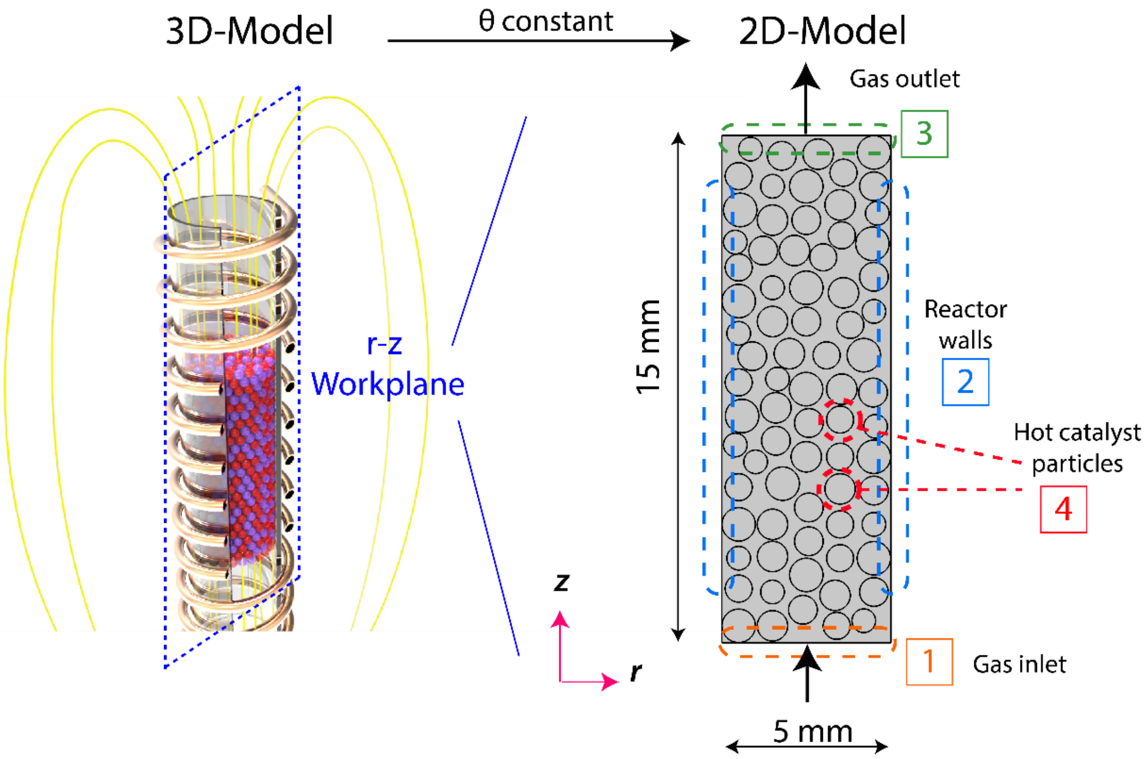

Figure EM2: Schematic view of the 2D geometric model developed for the CFD simulations to emulate in 2D the 3D packed-bed reactor inserted into a RF induction coil. The boundaries described in table EM1 and within the text are indicated as well.

1.3.2. Properties and operational parameters considered.

Physical and thermal properties for the multifunctional solid materials have been selected considering representative values for porous solids widely used as catalysts. Fluid properties were calculated for a diluted ethene stream (i.e. Ethene/Argon/Nitrogen 29.5/1.5/69 v/v) using Aspen Hysys v12.1, selecting the Peng-Robinson equation of state as property package. The properties were calculated at 1 bar and 673 K, which correspond to representative conditions for the simulated range of temperatures. A first set of simulations (CT1 and HS1), considering scenarios CT and HS, respectively, were run for a set of reference conditions as shown in table EM2. The results for these simulations are summarized in Figure 1d of the main text. Subsequently, a series of sensitivity studies were performed to evaluate the impact of different operational parameters on the average temperature difference between the "hot" and "cold" catalysts particles as described in section 1.3.3.

1.3.3. Sensitivity studies and average temperature gradient determination

A series of sensitivity studies considering the CT scenario were run, keeping constant the parameters selected for simulation CT1 and changing only one parameter at a time. The parameters screened were:

1. The Curie temperature of the *hot catalyst* particles (simulations CT1, CT2-CT4)
2. The wall-HTF global heat transfer coefficient U_{wall}, (simulations CT1, CT5-CT7)
3. The heat transfer fluid temperature, T_{HTF} (simulations CT1, CT8-CT10)
4. The gas space velocity or GHSV (simulations CT1, CT11-CT15)
5. The volume fraction of *hot catalyst* particles in the packed-bed (simulations CT1, CT16-CT17)

Analogously, a series of simulations were also performed to study the influence of various operational parameters for the high-temperature regime of the bed, considering a Curie temperature of the hot catalyst of 973 K (i.e. conditions of the simulation CT4). The parameters screened were:

1. The wall-HTF global heat transfer coefficient U_{wall}, (simulations CT4, CT18-C21)
2. The heat transfer fluid temperature, T_{HTF} (simulations CT4, CT22-CT24)
3. The gas space velocity or GHSV (simulations CT4, CT25-CT27)

Moreover, an additional sensitivity study was performed for the HS scenario, varying the power input at the magnetic suscepting particles of the *hot* catalyst (simulations HS1-HS5).

In all cases, the average temperature across the packed bed was individually determined for particles of the *hot* and *cold* catalysts, respectively, using the *"surface average"* option available in Comsol Multiphysics. The average temperature difference between the *hot* and *cold* catalysts was determined as the difference between said bed-averaged surface temperatures. All the simulation results are presented in Figures 1d of the main text and S1-S2 of this Supporting Information.

Table EM2. *Physical/thermal properties and operational parameters considered for the CFD simulations.*

Solids properties		
Property	***Hot catalyst***	***Cold catalyst***
Particle density, $\boldsymbol{\rho}$ (kg m^{-3})	750	750
Heat capacity ct P $\boldsymbol{C_p}$ (J Kg^{-1} K^{-1})	750	980
Thermal conductivity $\boldsymbol{k}$ (W m^{-1} K^{-1})	0.5	0.5
Fluid properties (determined at 1 bar and 673 K)		
Property	***Value***	
Fluid density, $\boldsymbol{\rho}$ (kg m^{-3})	0.6	
Heat capacity constant P $\boldsymbol{C_p}$ (J Kg^{-1} K^{-1})	1429	
Thermal conductivity $\boldsymbol{k}$ (W m^{-1} K^{-1})	4.6 x 10^{-2}	
Fluid viscosity $\boldsymbol{\mu}$ (Pa s)	2.6 x 10^{-5}	
Simulation parameters for reference CT1 and HS1 simulations		
Inlet gas temperature (K)	473	
HTF temperature (K)	373	
Global heat transfer coefficient Wall-to-HTF, $\boldsymbol{U_{wall}}$ (W m^{-2} K^{-1})	5000	
GHSV (h^{-1})	96350	
T Curie (K), only for CT scenario (K)	723	
RF heat source, only for HS scenario (W m^{-3})	6.6 x 10^{7}	
Volume fraction of *hot* catalyst (%)	40	

1.4. Catalytic conversion experiments

Catalytic testing experiments have been performed in a quartz tubular fixed-bed reactor, with an internal diameter of 7.8 mm, a wall thickness of 1.1 mm and a total length of 300 mm. In all cases, tests were carried out at 1 bar total pressure. The reactor was equipped with a polycarbonate safety shield as a passive safety element. Gases were fed into the system through several mass flow controllers (Bronkhorst), previously calibrated using a DEFENDER 520L volumetric flowmeter (MesaLabs). Downstream of the mass flow controllers, a pressure gauge (Swagelok, 0-5 bar) was used to monitor the pressure during the experiments.

In a first setting, the reactor is equipped with conventional, i.e., catalyst unspecific, convective energy input, leading to a standard isothermal operation. In this case, the reactor is wrapped by a heat tracing element (Briskheat, 156 W) connected to a temperature PID controller (ASCON K48 programable). The reading provided by a K-type thermocouple, placed at the axial center of the catalyst bed is used as feedback to the control loop.

In a second setting, the reactor is equipped with catalyst-specific energy input in a radiofrequency (RF) oscillating magnetic field. In this case, the RF field is generated by a Power Cube 90/200 magnetic field generator (CEIA, nominal maximum power 6 kW) operating at a frequency of 200 kHz and connected to a PWH-17-3-30/200 heating head (CEIA). The heating head is equipped with a custom-designed 5-looped hollow coil, (ID=38 mm, ED=50 mm and H=80 mm). Both units were internally cooled with a recirculated ethene glycol solution (50%, Repsol) provided by an external chiller (Julabo MODELFL4003) with a cooling power of 3 kW, and operating at a pumping pressure of 2 bar. The fraction of the maximum power delivered to the heating head can be adjusted by means of a controller (CEIA). Furthermore, the controller is connected to a switch equipped with an emergency safety shutoff button. Figure EM3 shows a schematic of the experimental setup. For RF heating experiments, average reactor wall temperatures were assessed with a pyrometer (DIAS PYROSPOT, model DGE 10NV, range 373-1573 K). Measuring parameters (i.e. transmissivity and emissivity) were calibrated by monitoring the cooling down process with an external type-K thermocouple connected to a temperature display. Calibration was performed using a reactor as the one described in section 1.2.8, inserting the thermocouple in the quartz sheath.

The influence of the operation temperature on the performance of the individual reactions in the tandem process was investigated under isothermal conditions.

In a typical ethene dimerization (ED) reaction test, 0.4 g of the Ni/Al-SiO_2 catalyst, previously sieved in the particle size range of 1-2 mm, were loaded as a packed bed on a quartz porous frit in the reactor, and diluted with silica (Davisil 710 NW, Grace), sieved in the same fraction, to reach a bed volume of 5 cm^3. Next, the catalyst was activated *in situ*, prior to reaction, under a flow rate of N_2 (Linde, 99.999%%, 80 mLN min^{-1}) at 1 bar by heating to 423 K for 6 h and 823 K for 2 h, using heating ramps of 5 K min^{-1}. Following catalyst activation, the reactor was first let cool down to RT and then it was heated up to the desired reaction temperature in the range 323-453 K at a heating rate of 10 K min^{-1} under flowing N_2. Subsequently, the nitrogen gas flow was switched to a feed mixture of 29.5/1.5/69 C_2H_4/Ar/N_2 (v/v) developed via mixing flows of N_2 and of a certified C_2H_4/Ar gas mixture (Linde, ethene/Ar 95/5 (v/v)), and the overall feed flowrate was adjusted to reach a WHSV of 1.1 g_{ethene} $g_{EDcatalyst}^{-1}$ h^{-1}.

In a typical olefin metathesis (OM) reaction test, 1.2 g of the Re/USY catalyst, previously sieved in the particle size range of 1-2 mm, were loaded as a packed bed on a quartz porous frit in the reactor, and diluted with silica (Davisil 710NW, Grace), sieved in the same fraction, to reach a

bed volume of 5 cm^3. Then, *in situ* catalyst activation was performed as detailed above for ED tests. Following catalyst activation, the reactor was first let cool down to RT and then it was heated up to the desired reaction temperature in the range 323-453 K at a heating rate of 10 K min^{-1} under flowing N_2. Subsequently, the nitrogen gas flow was switched to a flow of a certified gas mixture of 21/7/5/67 C_2H_4/1-butene/Ar/He (v/v) (Linde), and the overall feed flowrate was adjusted to reach a WHSV of 0.33 $g_{olefins}$ $g_{OMcatalyst}^{-1}$ h^{-1}.

In a typical tandem ethene dimerization/metathesis reaction test, preset masses of the multifunctional composite ED and OM materials, incorporating both catalytic and auxiliary functionalities, and previously sieved in the particle size range of 1-2 mm, were loaded as a packed bed on a quartz porous frit in the reactor. Next, *in situ* catalyst activation was performed under N_2 flow as detailed above. Then, the reactor was heated to the preset reaction temperature by means of either the heating element (catalyst-unspecific heating, isothermal operation) or axially inserted in the induction coil and subjected to the RF field (catalyst-specific heating). In the latter case, an input power for the RF device of 99 % was set for the first 10 minutes to heat up the system sufficiently fast and afterwards the power input was lowered to 80 % of the total power, until reaching a pseudo steady-state wall reactor temperature of ca. 393 K as determined with the pyrometer reading. Next, the setup for *operando luminescence thermometry* was assembled to attain catalyst-specific thermometry (*vide supra* section 1.2.7) and laser irradiation started. Finally, the nitrogen gas flow was switched to admit a flow of feed mixture of 29.5/1.5/69 C_2H_4/Ar/N_2 (v/v) into the reactor, and the feed flowrate was adjusted to reach a WHSV of 0.28 g_{ethene} $g_{catalysts}^{-1}$ h^{-1}. All catalyst-specific temperatures reported for the tandem catalytic experiments are those ones derived from the *operando luminescence* thermometry. The overall wall reactor pyrometer reading was used only as a guidance.

In all cases, the reactor's gas outlet was analyzed periodically in an Agilent 8860 gas chromatograph (GC), connected online, downstream of the reactor. In all experiments, transfer lines downstream of the reactor to the GC were heated at 393 K to avoid undesired product condensation. All heat tracing elements in the setup were thermally insulated to minimize heat losses. The GC is equipped with flame-ionization (FID) and thermal conductivity (TCD) detectors and used He as carrier gas in all analysis channels. Along a first analysis channel, leading to the FID, a DB-1 column (60 m length, 3 μm film thickness) was installed to resolve hydrocarbon compounds. Along a second analysis channel, leading to the TCD, a 10-port gas sampling valve was connected to a SP2100 PAW 80/100 precolumn, which allowed backflushing C_{3+} organic compounds. Next, a HP-Plot-Q (30 m, 20 μm) and a HP-Molesieve (30 m, 12 μm) capillary column, which stands either in series or bypass configurations downstream of the HP-Plot-Q column, were installed to resolve light compounds (C_2-C_4 hydrocarbons) and permanent gases (Ar and N_2), respectively. Product quantification was performed using chromatographic response factors

referenced to Ar as an internal standard. Gas-solid contact times were set to attain ethene conversions in the range of 5-15 % per reactor pass. Ethene ($C_2^=$) conversion rates, product selectivity, propene ($C_3^=$) formation rate and carbon balance have been calculated according to equations 7-12. In all cases, carbon balances closed to 98-102 %.

$$C_2H_4 conversion\ (X_{C_2^=}) = \left(1 - {\tilde{F}_{C_2^=\ outlet}}/{\tilde{F}_{C_2^=\ inlet}}\right) x\ 100\ (\ mol\%) \quad \text{Eq. 7}$$

$$MT\ conversion\ (X_{MT}) = \left({\tilde{F}_{C_3^=\ outlet}}/{\tilde{F}_{1C_4^=\ inlet} - \tilde{F}_{1C_4^=\ outlet}}\right) x\ 100\ (mol\ \%) \quad \text{Eq. 8}$$

$$Product\ selectivity\ (S_i) = \left(\frac{\widehat{F_{c,J}}}{\sum_j \widehat{F_{c,J}}}\right) x\ 100\ (wt\ \ \%) \quad \text{Eq. 9}$$

$$C_2^=\ conversion\ rate\ (-r_{C_2^=}) = \left(\tilde{F}_{C_2^=\ inlet} \times X_{C_2^=} / mol\ metal\right)\ (h^{-1}) \quad \text{Eq. 10}$$

$$C_3^=\ formation\ rate\ (r_{C_3^=}) = \left(\tilde{F}_{C_3^=\ outlet} / mol\ metal\right)\ (h^{-1}) \quad \text{Eq. 11}$$

$$Carbon\ balance\ = \left(\frac{\sum_j \hat{F}_{c,j\ ,reactor\ outlet}}{\sum_j \hat{F}_{c,j\ ,reactor\ inlet}}\right) x\ 100\ (\%) \quad \text{Eq. 12}$$

wherein $\tilde{F}_i$ corresponds to the molar flowrate of the compound *"i"* in mol min^{-1}, $\hat{F}_i$ corresponds to the mass flowrate of the compound *"i" in* g min^{-1}.

In the case of the olefin metathesis tests, catalyst deactivation has been assessed quantitatively. A first-order kinetics deactivation model was selected since it provided the best fit to the experimental data. The normalized activity parameter (*a*) was defined as the ratio of the propene formation rate, expressed per unit metal, ($r_{C_3^=}$) at any deactivation time (t) to the maximum $r_{C_3^=}$ (Eq.13). In all cases, *a*=1 and deactivation time was set to zero at the point at which the catalyst had undergone *in situ* activation and the metathesis activity was maximum. The corresponding deactivation constant (k_D) was determined from Eq.14, after linearization of the experimental data

according to Eq.15.[11] Figure EM4 illustrates the fitting for selected olefin metathesis tests at different temperatures.

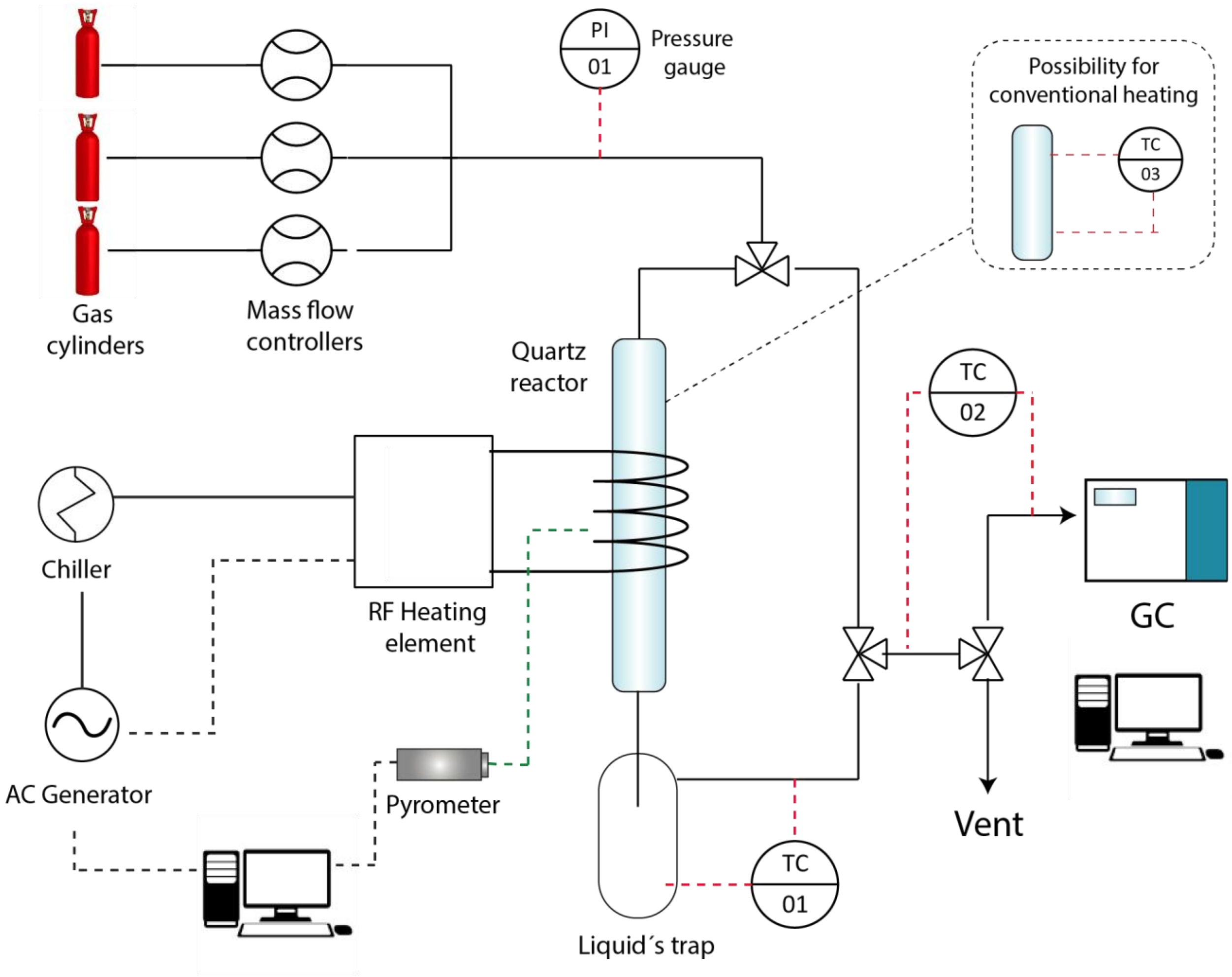

Figure EM3: Scheme of the reaction setup used for the catalyst-specific heating experiments, as described in section 1.4. The main figure shows the configuration employed for the experiments in which RF heating is used. On the top-right corner, the scheme that would be used for conventional convective heating (i.e. using a temperature controller connected to a heat tracing element) is shown.

$$a = \frac{r_{C_3^=}{}_{t=t}}{r_{C_3^=}{}_{t=max}} \qquad \text{Eq. 13}$$

$$-\frac{da}{dt} = k_D a \qquad \text{Eq. 14}$$

$$\ln a = \ln a_0 - k_D t \qquad \text{Eq. 15}$$

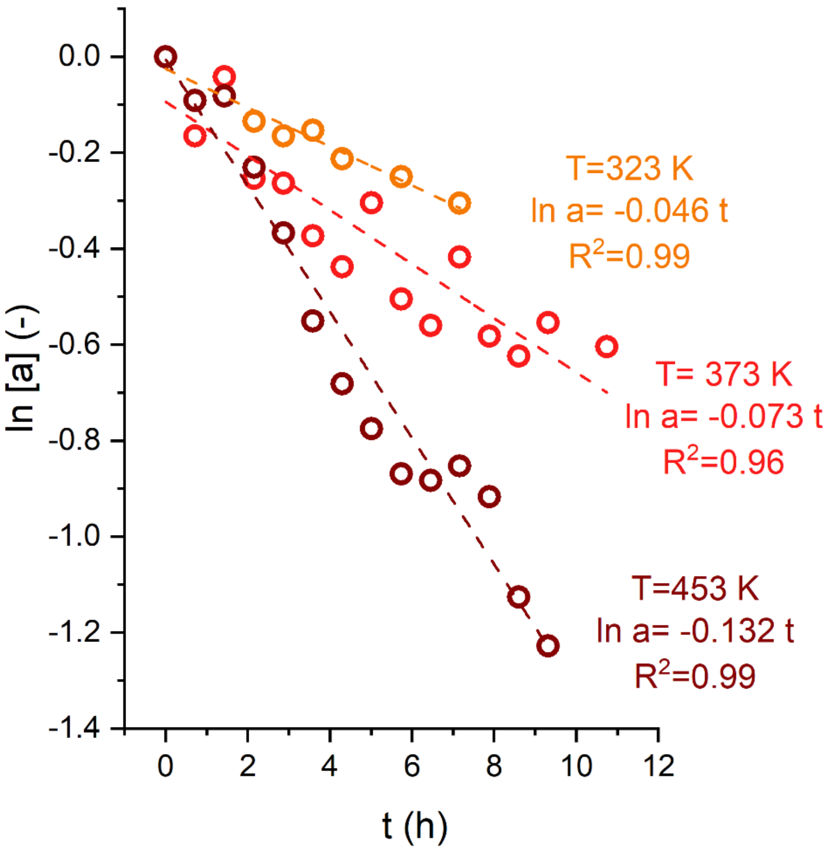

Figure EM4: Illustration of the fitting results to experimental data for the linearized first-order law describing the deactivation of the olefin metathesis (OM) Re/USY catalyst at reaction temperatures of 323, 373 and 453 K.

2. SUPPORTING FIGURES

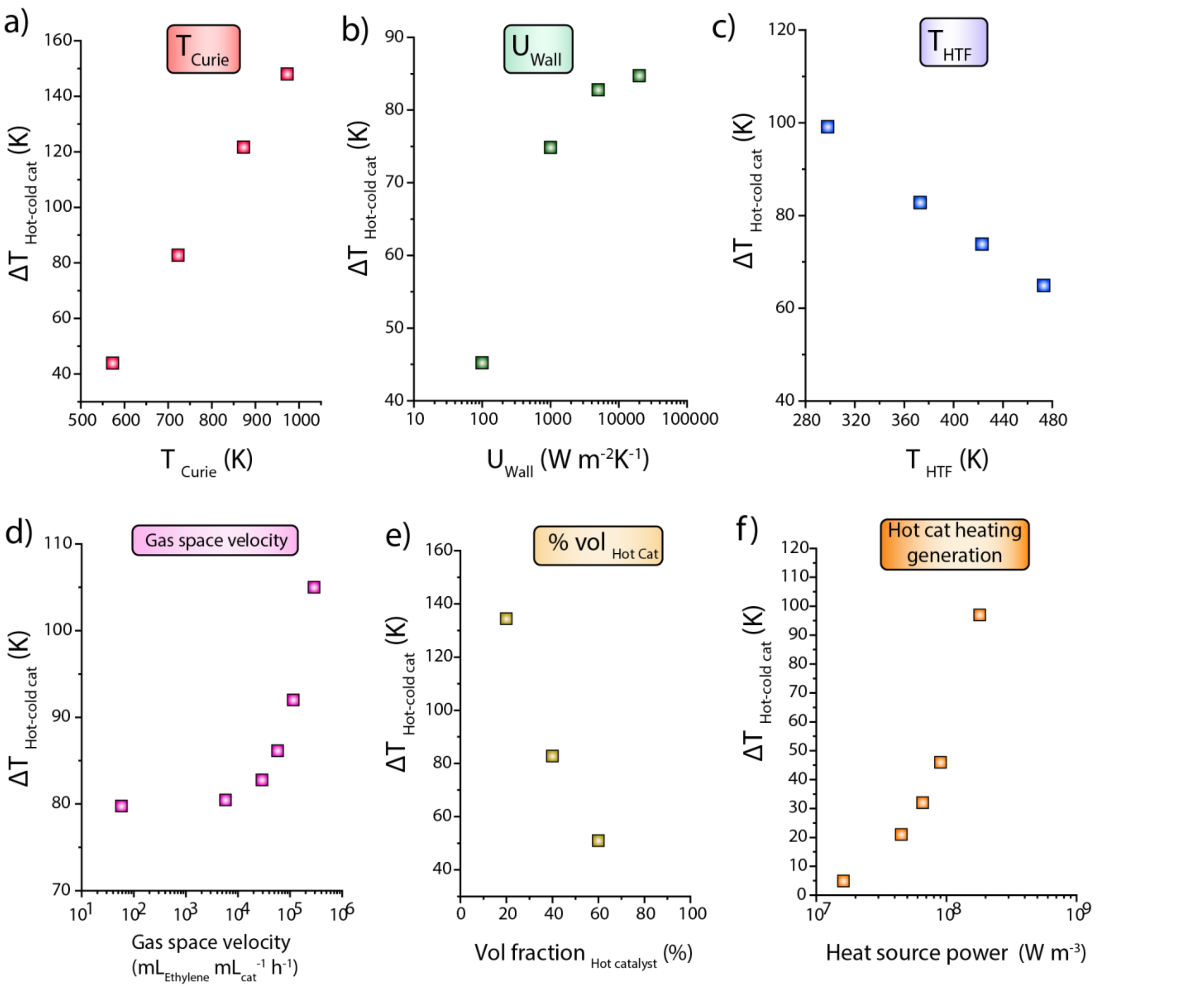

Figure S1: Evolution of the predicted steady-state average temperature gap between the *hot* and *cold* catalysts as a function of different system/operational parameters. The independent parameters screened correspond to: (a) the steady temperature of the hot catalyst particles assumed to operate isothermally at the Curie temperature of the magnetic nanosusceptors (CT1,CT2-CT4); (b) the overall heat transfer coefficient a the reactor's wall to the heat transfer fluid (CT1,CT5-CT7); (c) the temperature of the heat transfer fluid (CT1,CT8-CT10); (d) the gas space velocity along the packed bed (CT1,CT11-CT15); (e) the volume fraction of the *hot catalyst* in the packed bed and (CT1,CT16-CT17); (f) the heat generation power of *hot* catalyst particles. Those results shown in panels a-e correspond to the CT scenario, whereas simulations in panel f (HS1-HS5) correspond to the HS scenario (see section 1.3 in this Supporting Information for further details). In all cases, only one system/operational parameter was let vary at once, keeping the value for the rest of the parameters fixed at their default values (see table EM2 above).

As shown in **panel a**, the increase of the heating capacity by increasing the Curie temperature contributes to larger values of the average T gradient because of the higher driving force for heat transfer, which triggers enhanced heat removal via convective cooling (both

convective and external). Similar conclusions can be extracted from **panels b and c**, which also indicate that, when the heat transfer via external cooling is favored, either by increasing the heat transfer coefficient or reducing the coolant temperature, the average temperature gradient is increased.

Panel d shows the effect of different gas space velocities (i.e. higher gas flowrates at constant packed bed size). The results indicate that the temperature gap scales up with the gas flowrate, showing an exponential increase at sufficiently high space velocities (above 10^4 h^{-1}). This can be rationalized if it is considered that at higher gas velocities not only the gas contact time is reduced and hence the chances to heat up the *cold catalyst*, but also the flow regime transition takes place from laminar to turbulent. Hence, and considering that the gas phase acts as a "heat transfer intermediate" between both catalysts, we believe that at sufficiently high velocities, the effect of the lower contact time predominates over the heat transfer via gas convective cooling.

On the other hand, the results on **panel e** indicate that the lower the fraction of hot catalyst within the bed the higher the temperature gap. This could be explained if we consider once again the role of the gas as heat transfer intermediate. In this case, the lowest the fraction of hot particles in the bed, the lowest the gas phase temperature can be kept and hence the temperature gap that can be reached increases. Finally, results in **panel f** show the effect of the heat power generation of the *hot catalyst* on the average temperature gap (assuming the HS scenario described in section 1.3). The results show that, at sufficiently low heating power values (below 2 x 10^7 W m^{-3}), the heat generation cannot overcome the heat losses via convective gas/external cooling, thus leading to the presence of insignificant temperature gradients. Nevertheless, when the heating power is increased, the temperature gradient scales up with the latter, showing an analogous trend as the one indicated in panel a.

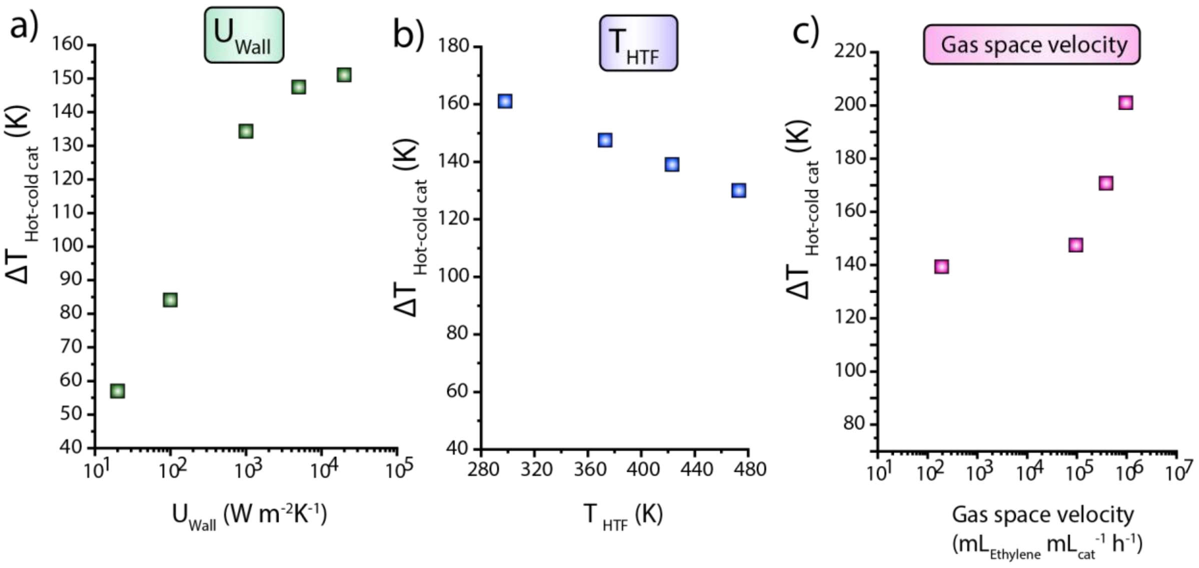

Figure S2: Evolution of the predicted steady-state average temperature gap between the hot and cold catalysts as a function of different system/operational parameters in the high temperature regime (T_{hot} catalyst =973 K). The independent parameters screened correspond to: (a) the overall heat transfer coefficient a the reactor's wall to the heat transfer fluid (CT4,CT18-CT21)); (b) the temperature of the heat transfer fluid (CT4,CT22-CT24); (c) the gas space velocity along the packed bed (CT4,CT25-CT27);

A series of CFD heat transfer simulations have been performed considering $T_{hot\ catalyst}$ =973 K, i.e. the case where the overall operation temperature regime of the packed bed is higher. In all cases, the CT model was adopted. Similarly, to the results shown in Figure S1 for lower overall temperature processes, different operational parameters have been varied to study their impact on the temperature difference between the hot and the cold catalyst. The results show in all cases analogous trends to those discussed for Figure S1, though attaining in all cases higher intercatalyst temperature differences. These observations can be rationalized if we consider that in this higher T regime, the contribution of the convective heat losses, through the reactor's walls, to the energy equation gains weight as a result of the higher driving force (temperature difference between the temperature of the system and the external/heat transfer fluid). Lower intercatalyst ΔT would still be feasible as shown in panel a, by designing cooling systems with lower overall heat transfer coefficients (in practice, exploiting for instance the low heat transfer capacity of natural convection systems).

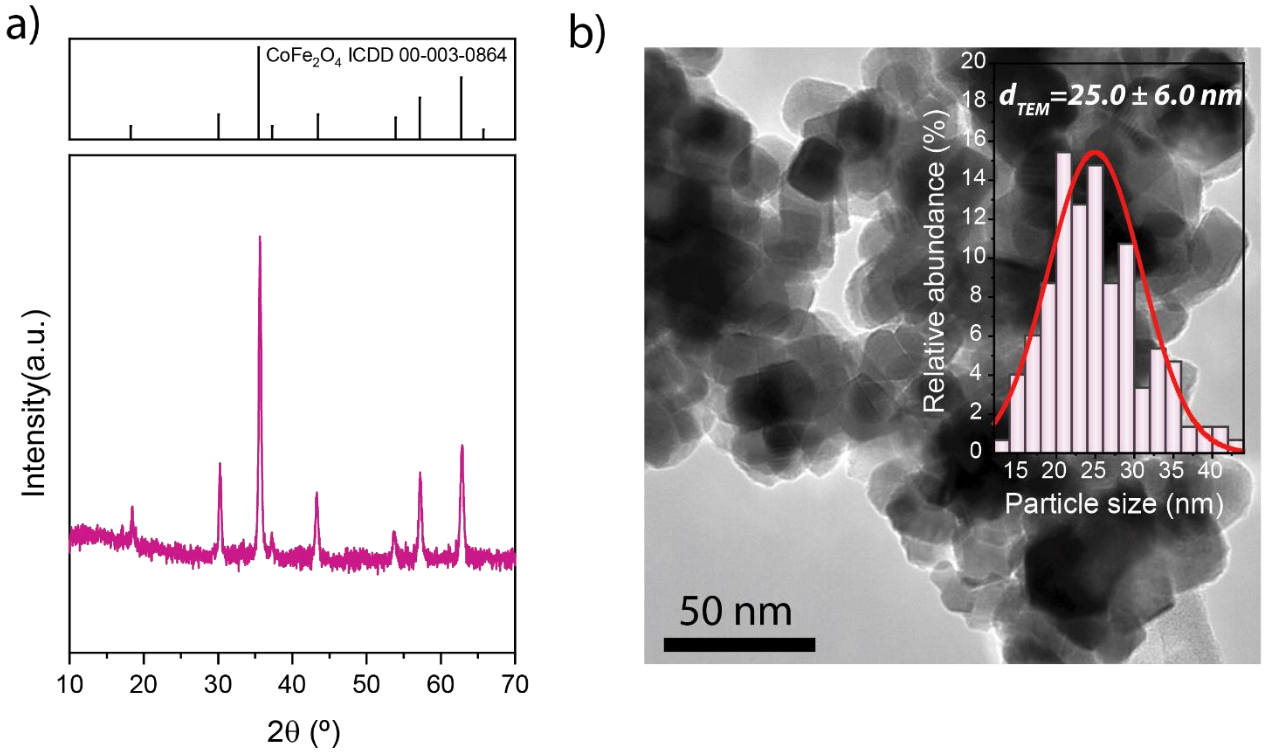

Figure S3: a) Powder XR diffractogram and b) bright-field TEM micrograph for $CoFe_2O_4$ ferrite nanocrystals after air calcination at 973 K (the scale bar is 50 nm). On panel (a), the corresponding diffraction lines for reference $CoFe_2O_4$ structure are included for comparison. Particle size distribution determined from bright-field TEM micrographs is incorporated as an inset. Values reported on the histogram inset correspond to the average particle size and the standard deviation.

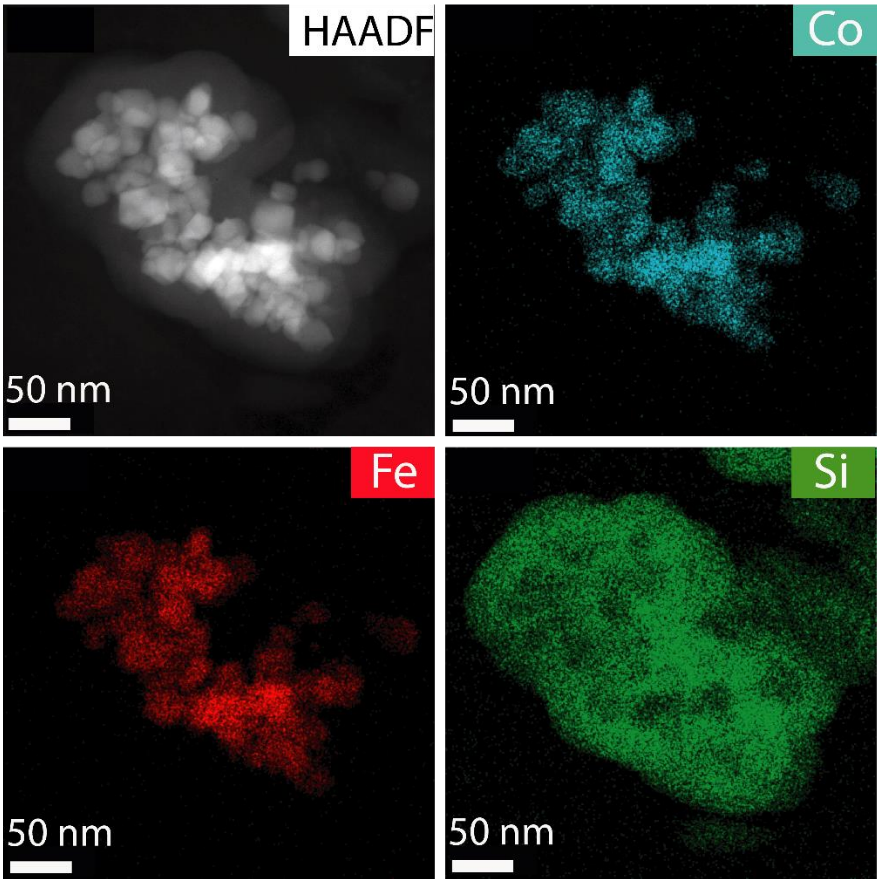

Figure S4: High Angle Annular Dark Field-Scanning Transmission Electron Microscopy (HAADF-STEM) and Energy Dispersive Spectroscopy (EDS) compositional maps for ultramicrotomed sections of $CoFe_2O_4@SiO_2$, after calcination at 973 K under stagnant air atmosphere.

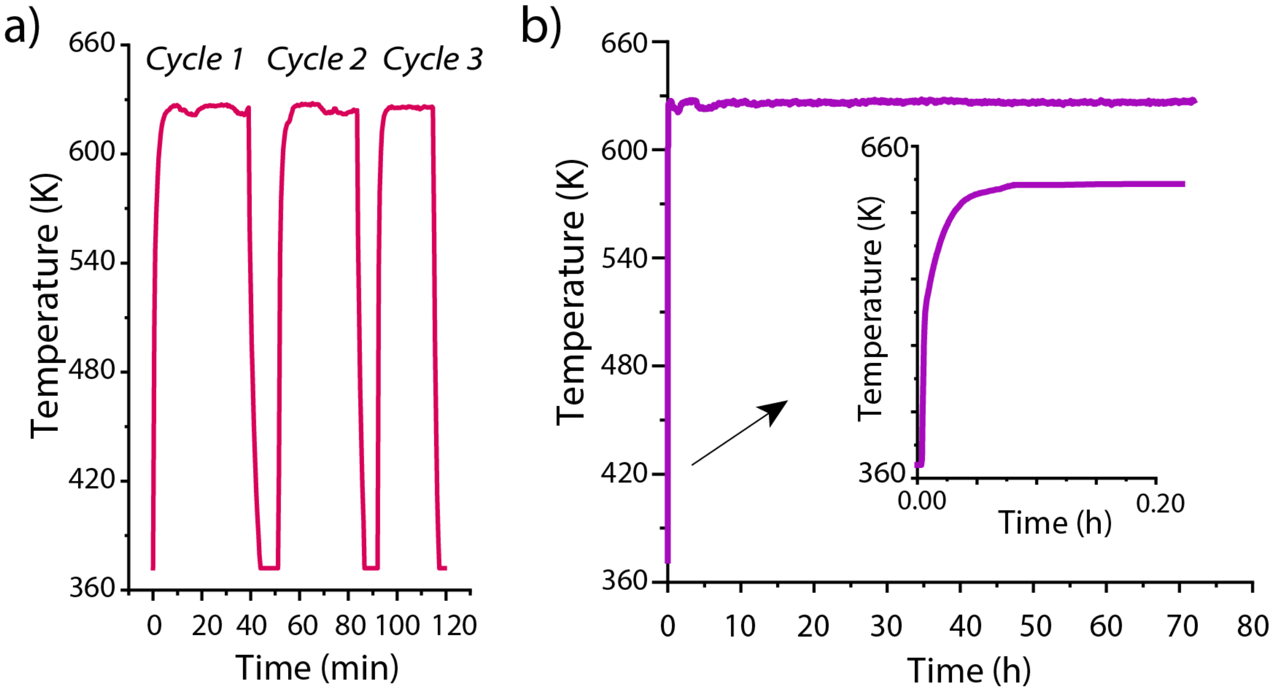

Figure S5: a) Heating-cooling operational cycles performed for the $CoFe_2O_4@SiO_2$ material under the RF field (200 kHz) at 99 % power output. b) Temperature stability test performed for the $CoFe_2O_4@SiO_2$ susceptors for more than 70 hours of operation time, with the initial heating rate shown as an inset in panel b.

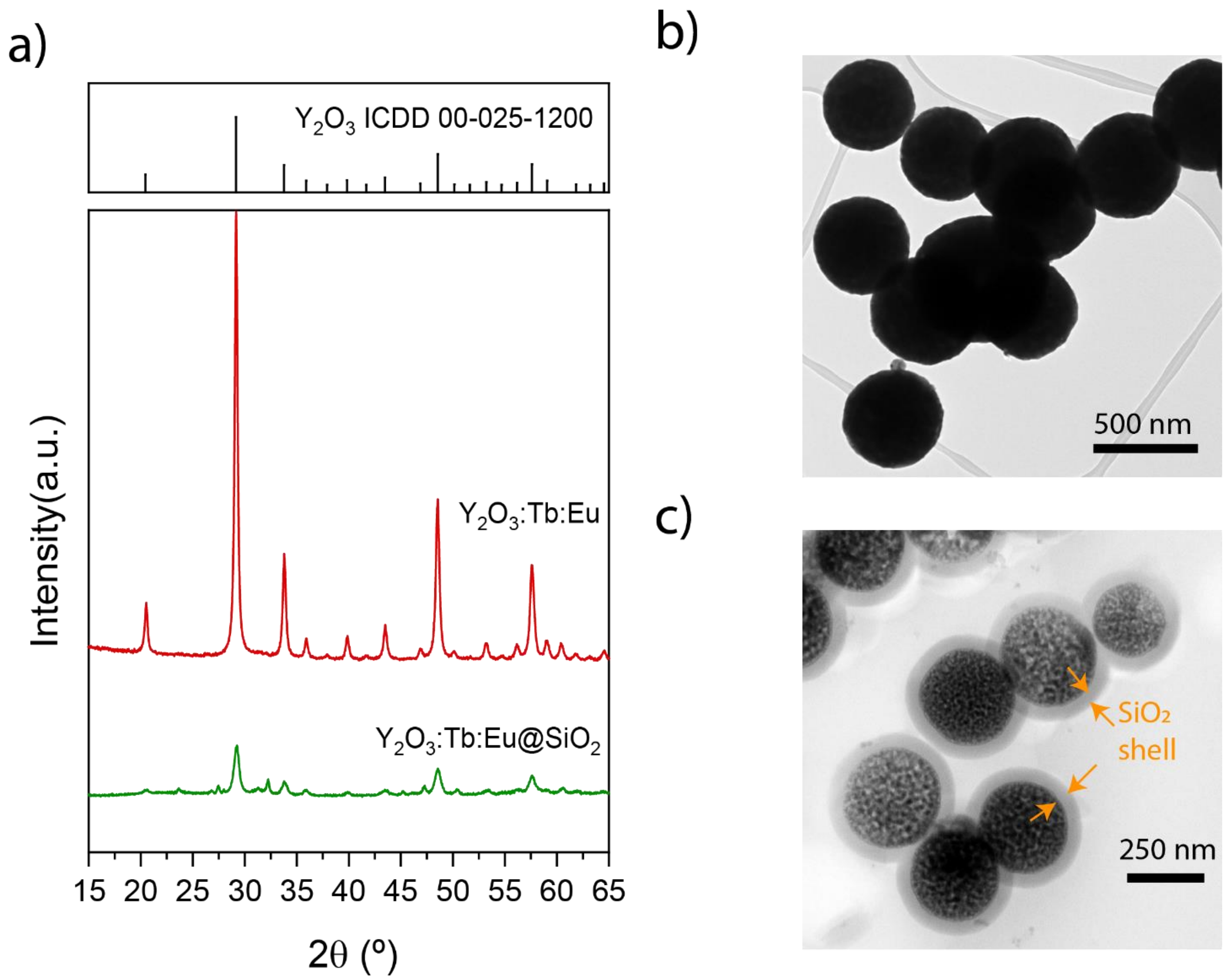

Figure S6: a) Powder XR diffractogram for Y_2O_3:Tb:Eu (-@SiO_2) after calcination at 973 K under stagnant air atmosphere; b) Bright-field TEM micrograph for Y_2O_3:Tb:Eu after air calcination at 973 K. c) High Resolution Bright-field TEM micrograph for ultramicrotomed sections of Y_2O_3:Tb:Eu@SiO_2 after air calcination at 973 K under stagnant air. On panel (a), the corresponding diffraction lines for reference Y_2O_3 structure are included for comparison.

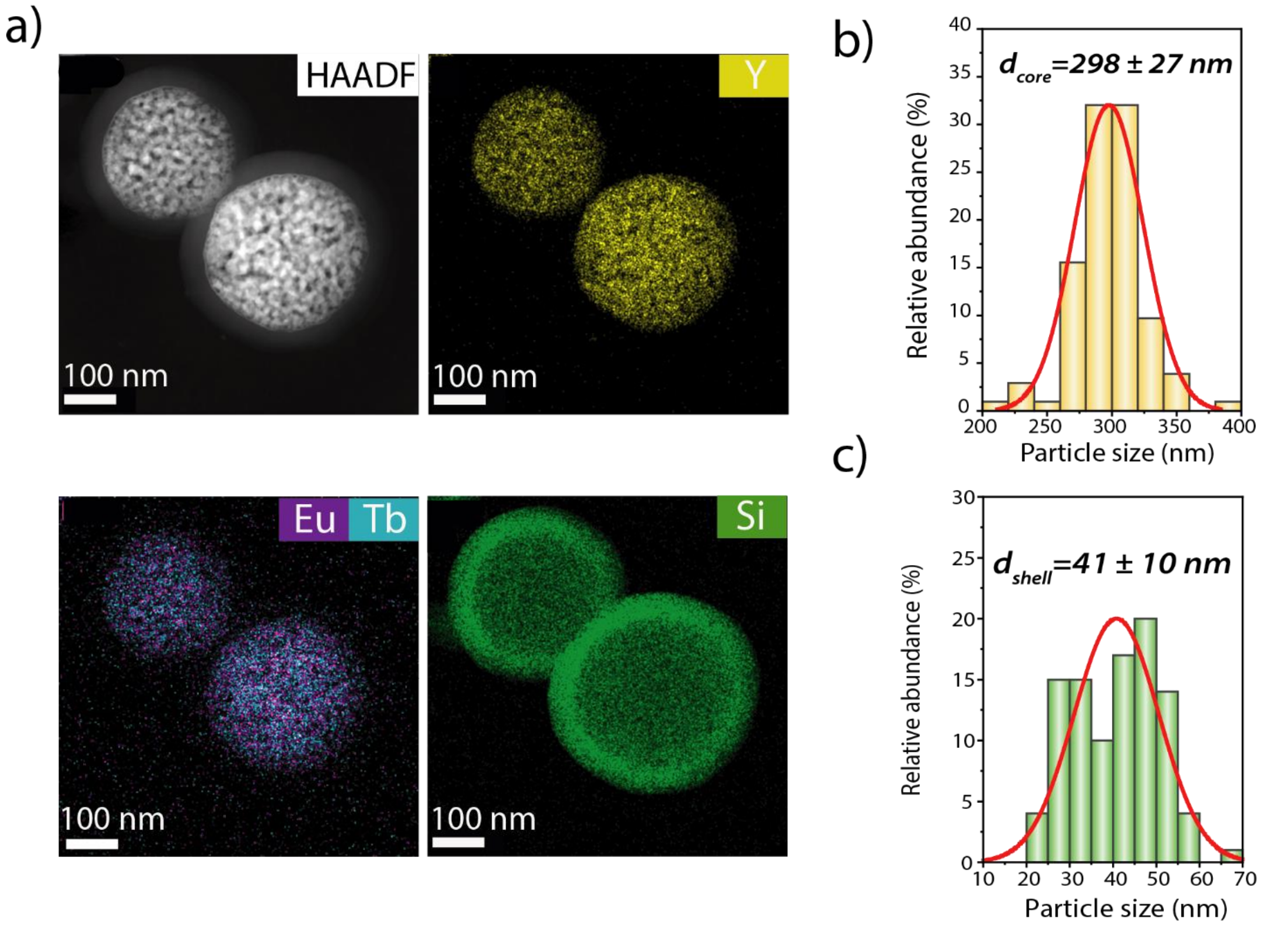

Figure S7: a) Representative High-Angle Annular Dark-Field Scanning Transmission Electron Microscopy (HAADF-STEM) and X-ray Energy Dispersive Spectroscopy (EDS) compositional maps for ultramicrotomed sections of Y_2O_3:Tb:Eu@SiO_2, after calcination at 973 K under stagnant air atmosphere. b) Size distribution for the Y_2O_3:Tb:Eu core and c) Thickness distribution for the SiO_2 shell. For both distributions, average core size/shell thickness and standard deviation values are provided on the histograms.

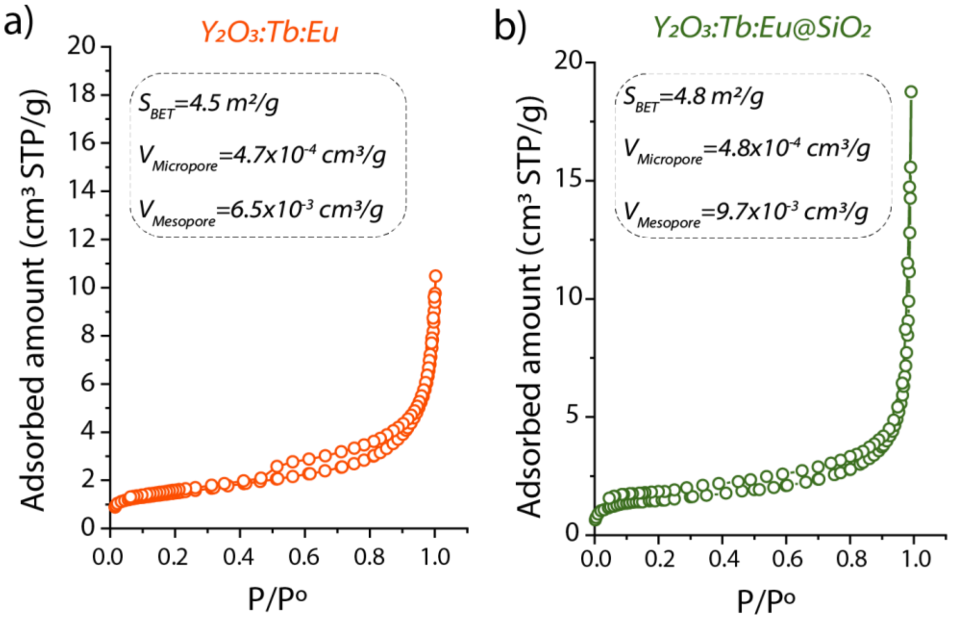

Figure S8: N_2 physisorption isotherms collected at 77 K for Y_2O_3:Tb:Eu (a) and silica-coated Y_2O_3:Tb:Eu@SiO_2 (b) phosphor nanomaterials, respectively. The insets to the plots indicate the BET specific surface area as well as the micropore and mesopore volumes determined from the analysis of the isotherms.

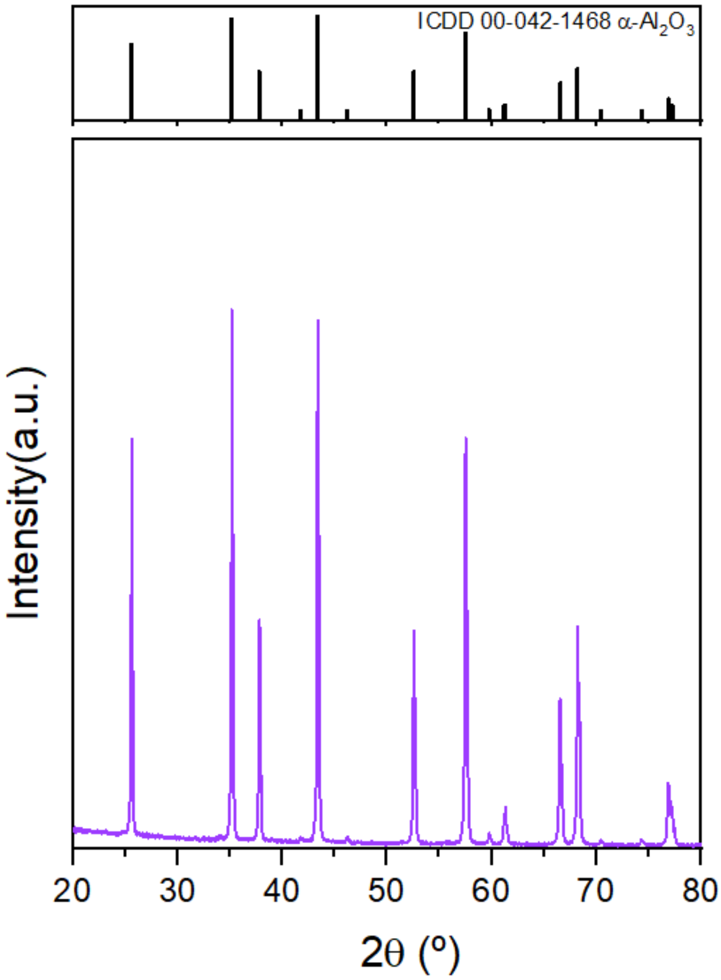

Figure S9: a) Powder XR diffractogram for α-Al_2O_3:Cr. On top, reference diffraction lines for α-Al_2O_3 are included for comparison.

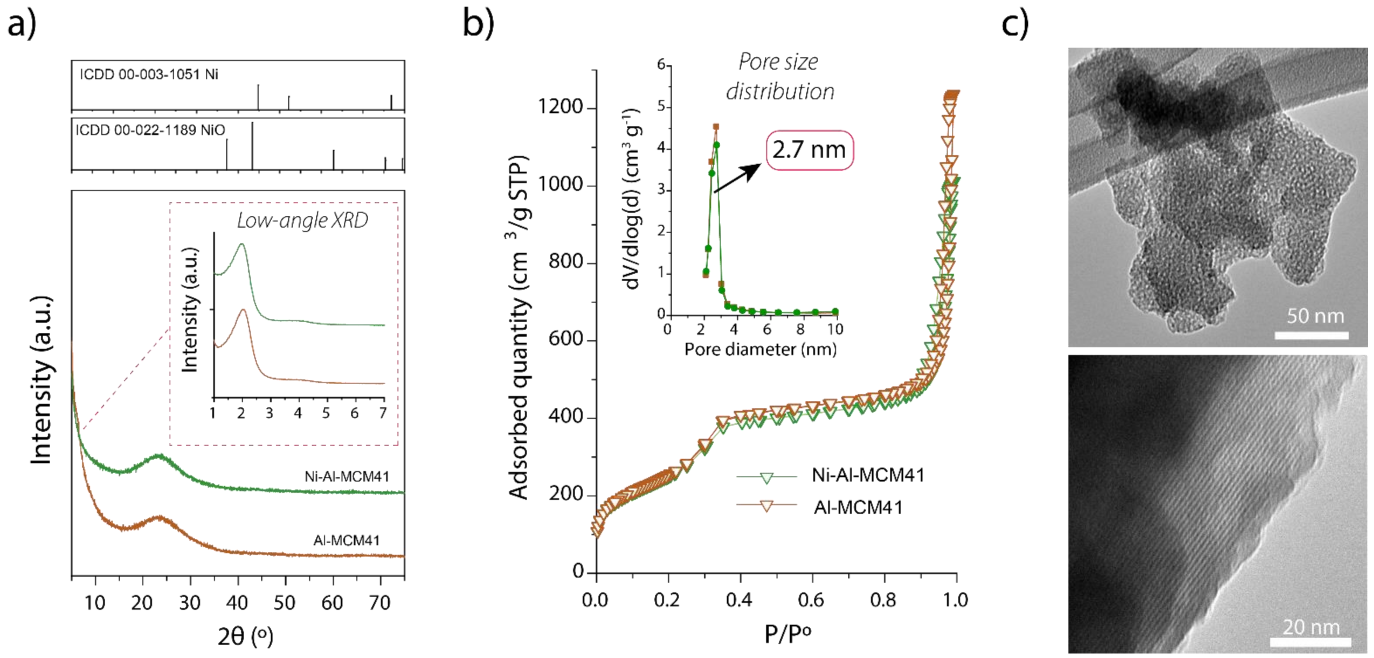

Figure S10: a) Powder XR diffractogram for Al-SiO_2 and Ni/Al-SiO_2. On top, reference diffraction lines for Ni and NiO are included for comparison. Low-angle XR powder patterns for Al-SiO_2 and Ni/Al-SiO_2 are displayed as an inset, showing low angle diffractions characteristic of mesoporous ordering. b) Nitrogen physisorption isotherms measured at 77 K for Al-SiO_2 and Ni/Al-SiO_2. The corresponding pore size distributions are also shown as an inset. c) Bright-field TEM micrographs for Ni/Al-SiO_2 samples.

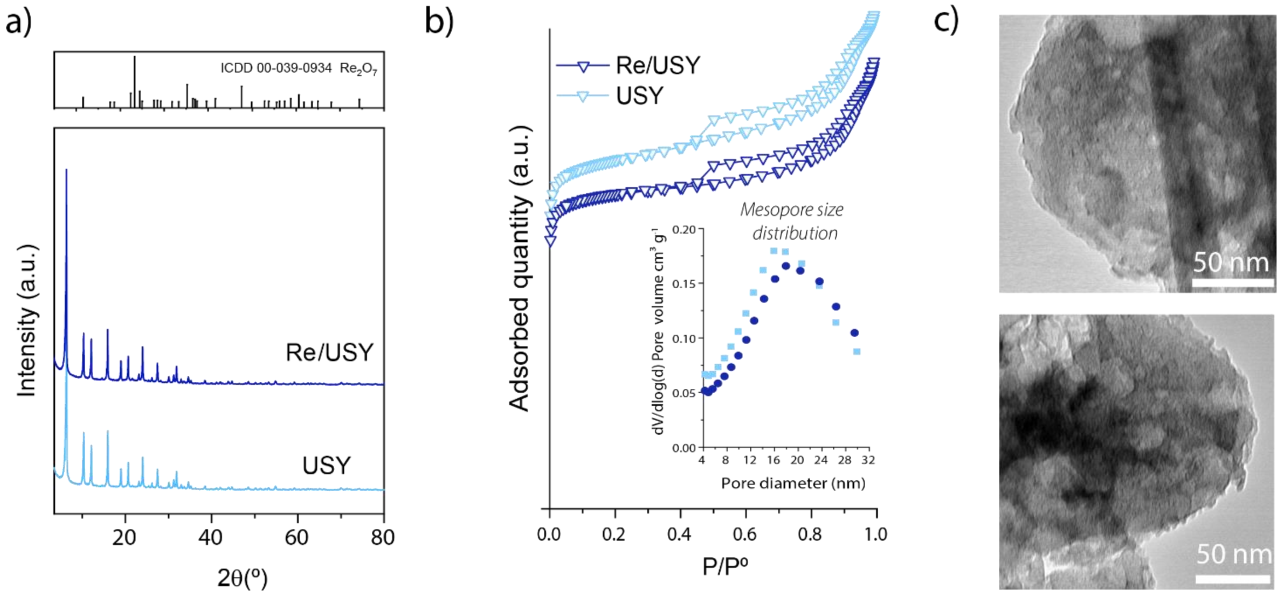

Figure S11: a) Powder X-ray diffractograms for the pristine USY catalyst support and the Re/USY olefin metathesis catalyst. On top, the corresponding diffraction lines for bulk Re_2O_7 are included to highlight the absence of rhenium oxide crystallites in Re/USY. **b)** N_2 physisorption isotherms and the corresponding mesopore size distributions (inset) for pristine USY and Re/USY. **c)** Representative bright-field TEM micrographs for Re/USY showing the mesoporous zeolite crystals.

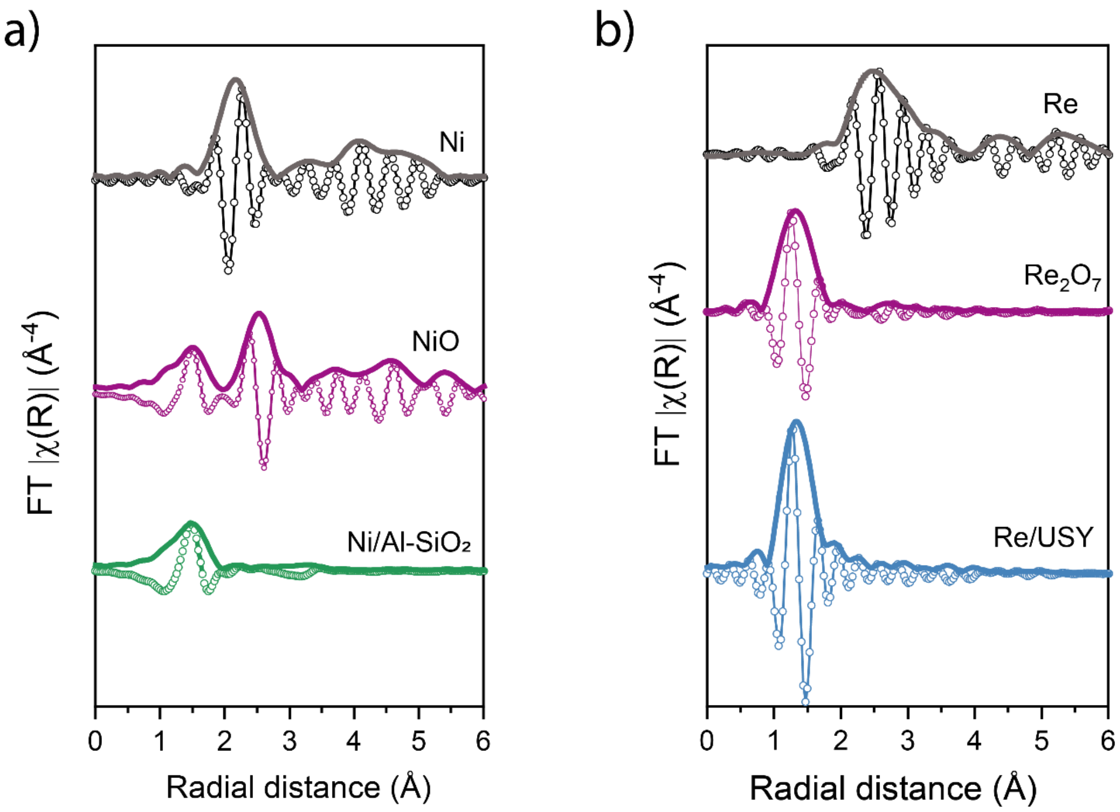

Figure S12: Fourier Transform of the k^3-weighted EXAFS function for a) Ni/Al-SiO_2 and b) Re/USY catalysts without phase correction, as shown in panels a and b respectively. The corresponding spectra for bulk nickel (II) oxide, rhenium (VII) oxide and metallic Ni (foil) and Re (powder) have been included for reference purposes.

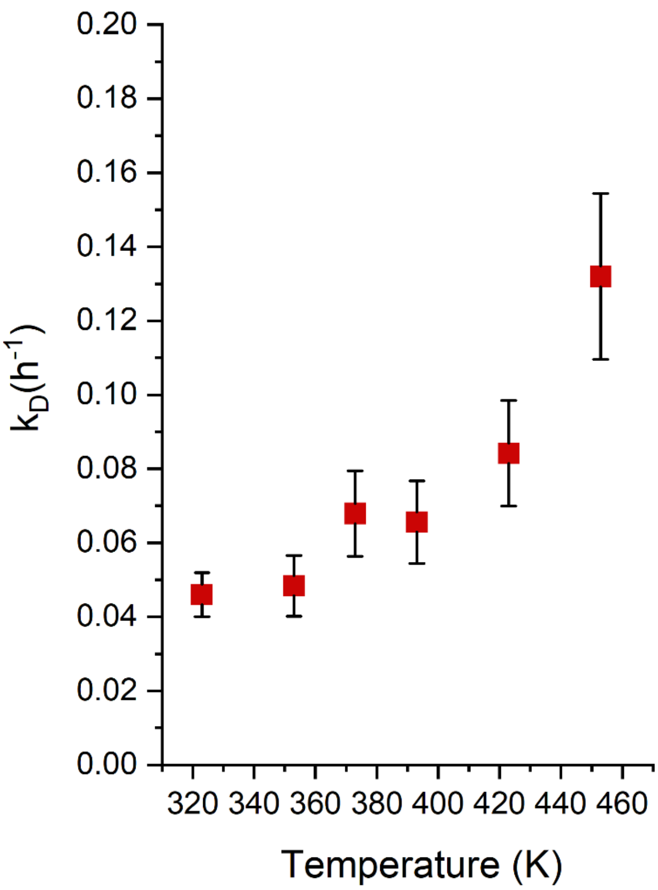

Figure S13: Olefin metathesis deactivation constants (k_D) as a function of the reaction temperature. The constants have been determined by assuming first-order deactivation kinetics (for further details see section 1.4 in this Supporting Information). Error bars correspond to the standard error of the mean (see equation 2).

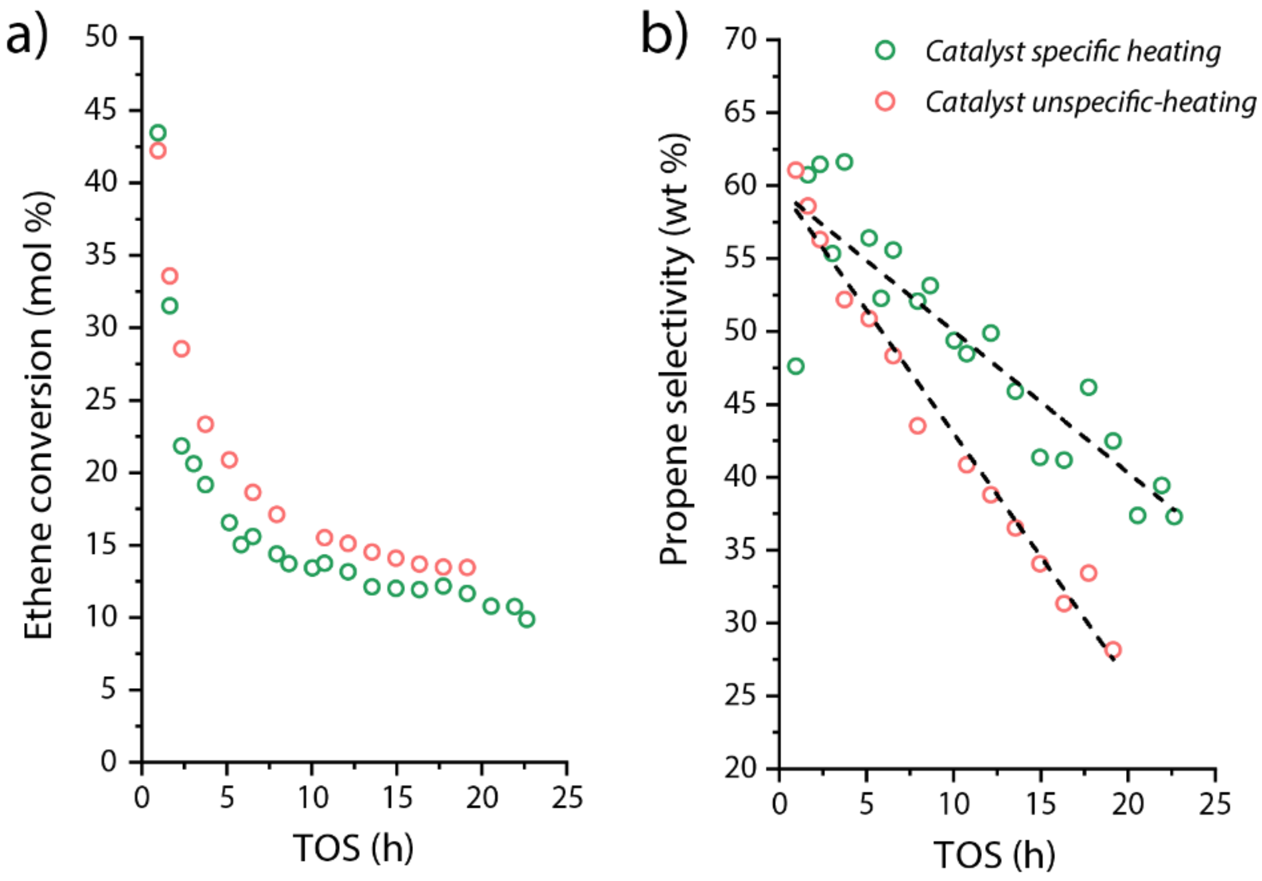

Figure S14: Evolution of the ethene conversion (a) and propene selectivity (b) as a function of time for the set of tandem dimerization/isomerization/metathesis conversion of ethene to propene experiments under conventional catalyst-unspecific (convective) heating and catalyst-specific (magnetic) heating, respectively.

3. SUPPORTING TABLES

Table S1: Chemical composition $CoFe_2O_4$ and $CoFe_2O_4@SiO_2$ nanomaterials as determined by ICP-OES.

Sample	Co (wt%)	Fe (wt%)	Si (wt %)
$CoFe_2O_4$	28.4	51.2	t.l.[a]
$CoFe_2O_4@SiO_2$	11.6	21.3	28.8

[a] Detected only at "trace levels".

Table S2. Chemical composition of Ni/Al-SiO_2 as determined by ICP-OES.

Sample	Si (wt%)	Al (wt%)	Ni (wt %)
Ni/Al-SiO_2	21.5	0.7	0.5

Table S3. Chemical composition of Re/USY as determined by ICP-OES.

Sample	Si (wt%)	Al (wt%)	Re (wt %)
Re/USY	34.7	6.4	2.9

4. REFERENCES

[1] A. Corma, V. Fornes, M. T. Navarro, J. Pérez Pariente, *J. Catal.* **1994**, *148*, 569–574.

[2] P. Zhao, L. Ye, G. Li, C. Huang, S. Wu, P.-L. Ho, H. Wang, T. Yoskamtorn, D. Sheptyakov, G. Cibin, A. I. Kirkland, C. C. Tang, A. Zheng, W. Xue, D. Mei, K. Suriye, S. C. E. Tsang, *ACS Catal.* **2021**, *11*, 3530–3540.

[3] A. L. SHA, H. RA, A. AA, A. T, A. H, *Int. J. Adv. Technol.* **2017**, *08*, DOI 10.4172/0976-4860.1000196.

[4] W. Stöber, A. Fink, E. Bohn, *J. Colloid Interface Sci.* **1968**, *26*, 62–69.

[5] J.-G. Li, X. Li, X. Sun, T. Ishigaki, *J. Phys. Chem. C* **2008**, *112*, 11707–11716.

[6] B. Ravel, M. Newville, *J. Synchrotron Radiat.* **2005**, *12*, 537–541.

[7] C. D. S. Brites, P. P. Lima, N. J. O. Silva, A. Millán, V. S. Amaral, F. Palacio, L. D. Carlos, *Nanoscale* **2012**, *4*, 4799.

[8] L. Zhong, S. Jiang, X. Wang, Y. Wang, J. Xie, Y. Li, B. Huang, Y. Wang, G. Xiang, L. Li, X. Zhou, M. Yin, *Inorg. Chem. Front.* **2022**, *9*, 5757–5765.

[9] S. Rebughini, A. Cuoci, M. Maestri, *Chem. Eng. Sci.* **2016**, *141*, 240–249.

[10] A. G. Dixon, M. Nijemeisland, E. H. Stitt, *Comput. Chem. Eng.* **2013**, *48*, 135–153.

[11] O. Levenspiel, *Chemical Reaction Engineering*, **1998**.
